# Chemotaxis of the Human Pathogen Pseudomonas aeruginosa to the Neurotransmitter Acetylcholine

**DOI:** 10.1128/mbio.03458-21

**Published:** 2022-03-07

**Authors:** Miguel A. Matilla, Félix Velando, Ana Tajuelo, David Martín-Mora, Wenhao Xu, Victor Sourjik, José A. Gavira, Tino Krell

**Affiliations:** a Department of Environmental Protection, Estación Experimental del Zaidín, Consejo Superior de Investigaciones Científicas, Granada, Spain; b Max Planck Institute for Terrestrial Microbiology and Center for Synthetic Microbiology (SYNMIKRO), Marburg, Germany; c Laboratory of Crystallographic Studies, IACT (CSIC-UGR), Armilla, Spain; University of California, Santa Cruz; The Ohio State University

**Keywords:** chemotaxis, chemoreceptor, *Pseudomonas aeruginosa*, acetylcholine, *Pectobacterium atrosepticum*, quaternary amines, neurotransmitter

## Abstract

Acetylcholine is a central biological signal molecule present in all kingdoms of life. In humans, acetylcholine is the primary neurotransmitter of the peripheral nervous system; it mediates signal transmission at neuromuscular junctions. Here, we show that the opportunistic human pathogen Pseudomonas aeruginosa exhibits chemoattraction toward acetylcholine over a concentration range of 1 μM to 100 mM. The maximal magnitude of the response was superior to that of many other P. aeruginosa chemoeffectors. We demonstrate that this chemoattraction is mediated by the PctD (PA4633) chemoreceptor. Using microcalorimetry, we show that the PctD ligand-binding domain (LBD) binds acetylcholine with a equilibrium dissociation constant (*K_D_*) of 23 μM. It also binds choline and with lower affinity betaine. Highly sensitive responses to acetylcholine and choline, and less sensitive responses to betaine and l-carnitine, were observed in Escherichia coli expressing a chimeric receptor comprising the PctD-LBD fused to the Tar chemoreceptor signaling domain. We also identified the PacA (ECA_RS10935) chemoreceptor of the phytopathogen Pectobacterium atrosepticum, which binds choline and betaine but fails to recognize acetylcholine. To identify the molecular determinants for acetylcholine recognition, we report high-resolution structures of PctD-LBD (with bound acetylcholine and choline) and PacA-LBD (with bound betaine). We identified an amino acid motif in PctD-LBD that interacts with the acetylcholine tail. This motif is absent in PacA-LBD. Significant acetylcholine chemotaxis was also detected in the plant pathogens Agrobacterium tumefaciens and Dickeya solani. To the best of our knowledge, this is the first report of acetylcholine chemotaxis and extends the range of host signals perceived by bacterial chemoreceptors.

## INTRODUCTION

Chemotaxis allows bacteria to move in chemical concentration gradients and facilitates the colonization of more-favorable ecological niches. Genome analyses indicate that about half of known bacterial species possess genes required for chemotaxis (1). The molecular machinery for chemotaxis is highly complex and is among the best-studied bacterial signal transduction systems. Chemoeffectors are sensed by chemoreceptors that in turn stimulate chemosensory pathways that modulate the activity of the flagellar motor (2, 3).

A large number of different chemoeffectors have been identified (4). Whereas some chemoeffectors serve as nutrients, including organic acids, amino acids, and sugars, other chemoeffectors provide information about the environment, as exemplified by chemotaxis to neurotransmitters (5), quorum-sensing signals (6), human hormones (7), and plant signaling molecules (8). Other chemoeffectors may have multiple functions, such as gamma-aminobutyrate (GABA) (9) and histamine (10), which are central signal molecules but also support bacterial growth.

A canonical chemoreceptor is composed of an extracytosolic ligand-binding domain (LBD) and a cytosolic region that contains the signaling domain that interacts with other signaling proteins. Whereas the signaling domain is highly conserved in sequence, there is an enormous variety in the LBD type and sequence (11). More than 80 different LBD types have so far been identified in chemoreceptors (11), and new LBD families continue to be discovered (12). The close correspondence in the affinities of full-length receptors and isolated LBDs (13–15) indicates that all of the features necessary for chemoeffector recognition are contained within the LBD.

The chemotactic machinery represents an important metabolic burden to the cell. For example, the synthesis of the chemotaxis system and assembling and energizing the flagellar motors in Escherichia coli consumes several percent of the total cellular protein and energy budget (16–18). Another study has shown that the removal of the 70-kb flagellar operon from Pseudomonas putida resulted in several physiological advantages and increased fitness (19). This considerable metabolic burden has to be compensated by major benefits arising from chemotaxis. However, our understanding of these benefits for bacteria with different lifestyles is currently very limited, largely because the signals recognized by the majority of chemoreceptors are unknown.

The chemoeffector repertoire of a bacterium is a reflection of its lifestyle (20, 21). Species that inhabit a specific ecological niche contain a reduced number of chemoreceptors. For example, Helicobacter pylori infects the gastric epithelium and is adapted to a highly specific niche. This species has 4 chemoreceptors, which is a number significantly below the bacterial average of 14 (1). Analysis of H. pylori chemoreceptor function has revealed a specialized spectrum of chemoeffectors that is closely linked to establishing an infection in the stomach. Furthermore, all four chemoreceptors were found to play a role in the infection process (11, 22–24).

In contrast, bacteria with a versatile lifestyle that are able to survive in different ecological niches have a much higher number of chemoreceptors (21). The opportunistic pathogen Pseudomonas aeruginosa serves as a model organism to study this category of bacterium. P. aeruginosa is omnipresent in the environment and has been detected in soil, water, human- and animal-derived samples, and different foods, including vegetables and milk, and in plumbing systems and hospitals (25–27). P. aeruginosa is also a highly versatile pathogen, able to infect almost all human tissues, including the respiratory tract, ear, eye, brain, heart, and urinary tract, and it can cause general bacteremia (28). P. aeruginosa is of great clinical relevance: (i) infections are associated with significant mortality, (ii) it is among the most frequent causes of nosocomial infections, and (iii) multidrug-resistant strains are rapidly emerging (29–32). The ubiquity of this pathogen is also reflected in its capacity to infect different animals and plants (33, 34). Considering its omnipresence in the environment and the versatility of its lifestyle, it is of high importance to identify the environmental signals that are sensed by the P. aeruginosa chemoreceptors.

P. aeruginosa has 26 chemoreceptors that stimulate four different chemosensory pathways (35). Whereas 23 chemoreceptors were predicted to stimulate the chemotaxis pathway (36), a single receptor communicates with each of the remaining three pathways that carry out functions unrelated to chemotaxis (35). A significant number of P. aeruginosa chemoreceptors have been functionally annotated. Among these are PctA, PctB, and PctC, which are involved in chemotaxis to different proteinogenic amino acids and GABA (37–39); CtpL and CtpH, which sense inorganic phosphate (40, 41); TlpQ, which senses histamine and polyamines (10); McpK, which senses α-ketoglutarate (42); McpN, which senses nitrate (43); CtpM, which senses malate and other C4 dicarboxylic acids (44, 45); CttP, which senses chloroethylenes (46); and Aer, which is involved in aerotaxis (47). Furthermore, PctA and TlpQ were found to bind and mediate chemoattraction to the autoinducer-2 quorum sensing signal (48). The dCache domain is the predominant extracellular LBD in bacterial signal transduction systems, and it is present in about 15% of all chemoreceptors (1, 49). P. aeruginosa has five chemoreceptors that contain dCache LBDs, four of which—PctA, PctB, PctC, and TlpQ—have been functionally annotated (35).

Here, we show that the fifth dCache domain-containing chemoreceptor, PA4633, binds the human neurotransmitter acetylcholine and induces a very strong attractant response to this chemoeffector. High-resolution three-dimensional (3D) structures of its LBD and of a homologous domain that does not bind acetylcholine reveal the determinants for acetylcholine recognition. Acetylcholine chemotaxis has been observed in other bacteria, and its functional relevance is discussed.

## RESULTS

### Chemotaxis response of P. aeruginosa PAO1 to acetylcholine.

**FIG 1:**
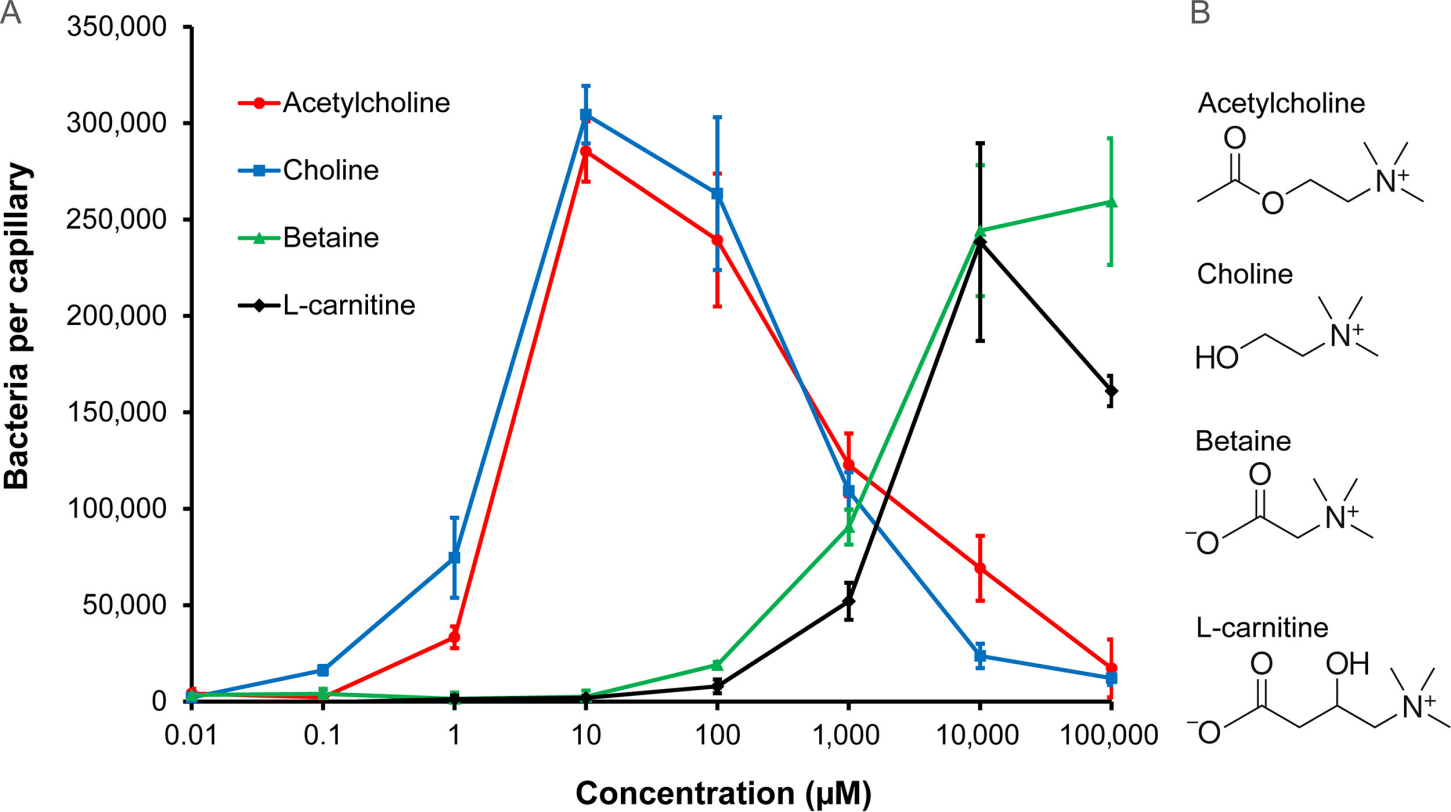
Chemotaxis of P. aeruginosa PAO1 toward acetylcholine and related compounds. (A) Quantitative capillary chemotaxis assays. Data have been corrected for the number of bacteria (7,825 ± 623) that swam into buffer-containing capillaries. Data are the means and standard deviations from three independent experiments conducted in triplicate. (B) The structures of the chemoeffectors.

The attraction to growth substrates is a major biological function of bacterial chemotaxis (20). To identify the compounds that support the growth of P. aeruginosa, we conducted 96-well plate assays in which we screened P. aeruginosa growth in minimal medium supplemented with the compounds from different Biolog arrays. As shown in Fig. S1A, significant growth was observed for l-carnitine. To assess whether P. aeruginosa performs chemotaxis to l-carnitine, we conducted quantitative capillary chemotaxis assays that revealed chemoattraction (Fig. 1).

10.1128/mbio.03458-21.1 FIG S1 Growth experiments with P. aeruginosa PAO1. Growth in M9 minimal medium supplemented with each of the compounds present in the Biolog compound array PM2A comprising carbon sources. Experiments were conducted in 96-well plates. The compounds that permit strongest growth are labelled. d,l-carnitine is the only quaternary amine in this compound array. The observation that d,l-carnitine permits growth as a carbon source has prompted experiments to determine whether it is a chemoeffector. Data represent growth for 48 h. Cells were grown at 37°C using a Bioscreen microbiological growth analyzer (Oy Growth Curves Ab Ltd., Helsinki, Finland) under continuous shaking. Download FIG S1, JPG file, 0.3 MB. Copyright © 2022 Matilla et al. 2022 Matilla et al. https://creativecommons.org/licenses/by/4.0/ This content is distributed under the terms of the Creative Commons Attribution 4.0 International license.

Responses were characterized by a rather high threshold of the response at 100 μM and a maximal response at 10 mM. We subsequently conducted experiments to find out whether structurally related compounds, such as other quaternary amines, also induced chemotaxis. We observed chemoattraction to betaine that was comparable to that of l-carnitine (Fig. 1). Importantly, choline and acetylcholine also induced chemotaxis (Fig. 1). In contrast to l-carnitine and betaine, responses to choline and acetylcholine occurred at much lower concentrations, with a response threshold of 0.1 μM for choline and 1 μM for acetylcholine. These response thresholds were at lower concentrations than for a number of other P. aeruginosa chemoeffectors analyzed with the same technique (40, 43, 44). Maximal responses induced by choline and acetylcholine were observed at 10 μM, and the maximal accumulation of ∼300,000 bacteria per capillary was significantly higher than the maximal accumulations in response to inorganic phosphate (40), nitrate (43), malate (44), and α-ketoglutarate (42). The large magnitude of the chemotaxis response acetylcholine evokes, the low threshold concentration, and its important physiological role as a neurotransmitter motivated studies to identify the corresponding molecular mechanism of acetylcholine sensing.

### Role of PctD (PA4633) in chemotaxis to acetylcholine.

The McpX chemoreceptor in Sinorhizobium (Ensifer) meliloti has been reported to mediate chemotaxis to quaternary amines (50). This chemoreceptor contains a dCache_1 type LBD. Of the P. aeruginosa chemoreceptors with dCache_1 LBDs, only PA4633 was of unknown function (35). The LBD of this receptor shares only 17% sequence identity with the McpX-LBD (Fig. S2A). To assess the role of PA4633 in chemotaxis to the four chemoeffectors, we have conducted quantitative capillary assays using a mutant defective in this chemoreceptor (Fig. S3). Apart from some minor responses to high concentrations of betaine, the *pp4633* mutant failed to respond to these four quaternary amines—phenotypes that were reversed to wild-type responses by the in *trans* expression of *pa4633* using a pBBR1MCS-based vector (Fig. S3E). Taken together, these data indicate that PA4633 is the sole chemoreceptor that mediates chemotaxis to these four quaternary amines at physiologically relevant concentrations. Chemoreceptor PA4633 was thus renamed PctD (Pseudomonas chemotaxis transducer D).

10.1128/mbio.03458-21.2 FIG S2 (A to C) Sequence alignments of the ligand-binding domains of P. aeruginosa PAO1 chemoreceptor PctD (PA4633) with S. meliloti McpX (A), *P. atrosepticum* SCRI1043 ECA_RS05475 (B), and *P. atrosepticum* SCRI1043 chemoreceptor ECA_RS10935 (PacA) (C). The alignment was done using the CLUSTALW algorithm of the NPS@ suite (107) using the GONNET weight matrix, a gap opening penalty of 10, and a gap extension penalty of 0.2. Red, identical; green, highly similar; blue, weakly similar. The amino acids that interact in the PctD-LBD and PacA-LBD structures with the bound ligand are shaded in yellow. Download FIG S2, JPG file, 1.1 MB. Copyright © 2022 Matilla et al. 2022 Matilla et al. https://creativecommons.org/licenses/by/4.0/ This content is distributed under the terms of the Creative Commons Attribution 4.0 International license.

10.1128/mbio.03458-21.3 FIG S3 (A to E) Quantitative capillary chemotaxis assays of P. aeruginosa PAO1, a mutant in *pa4633* (*pctD*) (A to D) and the mutant in *pa4633* harboring different plasmids for complementation (E). In panels A to D, data have been corrected with the number of bacteria (7,825 ± 623) that swam into buffer-containing capillaries. In panel E, the chemoeffector concentration was 100 μM. Strains were complemented with the empty plasmid pBBR1MCS-2_Start and its derivative containing the PA4633 encoding sequence (pMAMV347). Data have been corrected with the number of bacteria that swam into buffer-containing capillaries, namely, 23,137 ± 5,179 (PAO1 [pBBR1MCS2_START]), 16,400 ± 2,969 (PAO1-PA4633 [pBBR1MCS2_START]), and 8,362 ± 194 (PAO1-PA4633 [pMAMV347]). Data are the means and standard deviations from three independent experiments conducted in triplicate. Download FIG S3, JPG file, 0.6 MB. Copyright © 2022 Matilla et al. 2022 Matilla et al. https://creativecommons.org/licenses/by/4.0/ This content is distributed under the terms of the Creative Commons Attribution 4.0 International license.

### Effect of acetylcholine binding on the activity of PctD.

**FIG 2:**
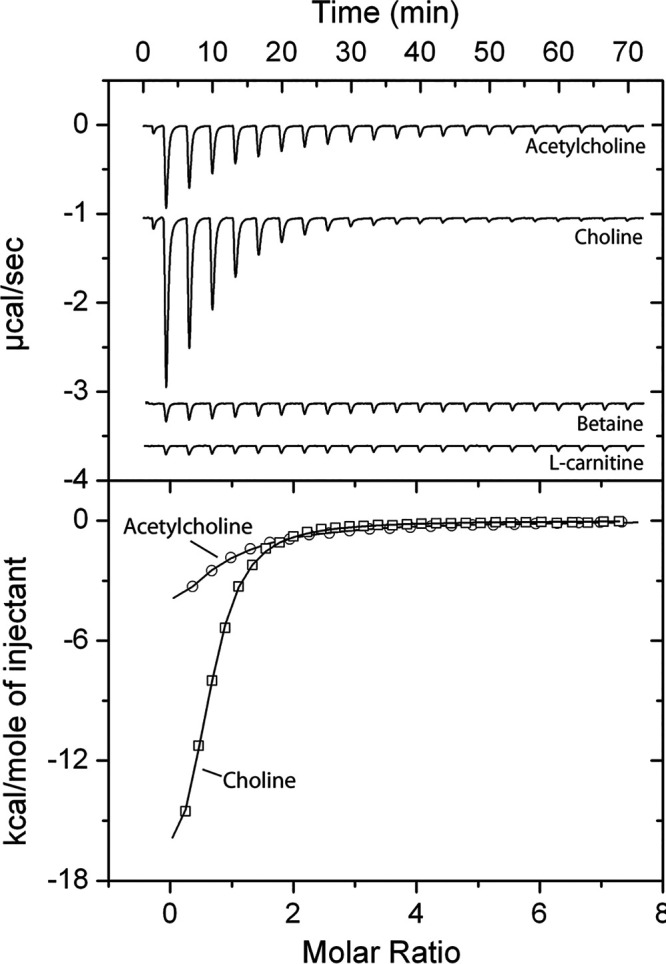
Isothermal titration calorimetry study of the binding of different ligands to the ligand-binding domain of chemoreceptor PctD. (Upper panel) Raw data for the titration of 15 to 22 μM protein with 9.6-μL aliquots of 0.5 to 10 mM ligand solutions. (Lower panel) Concentration-normalized and dilution heat-corrected raw data for the titration with acetylcholine and choline. The continuous line is the best fit with the “one binding site model” of the MicroCal version of ORIGIN.

There are a number of ways by which chemoeffectors stimulate chemoreceptors (11). To verify whether the chemoeffectors activate PctD by direct binding, the LBD of PctD was produced as purified recombinant protein and submitted to microcalorimetric binding studies. Acetylcholine and choline bound with high affinity, with dissociation constants of 23 and 2.6 μM, respectively (Fig. 2, Table 1). Binding of betaine occurred with a much lower affinity (*K_D_* = 990 μM). No response was observed with l-carnitine. This compound probably also binds, but the sensitivity of isothermal titration calorimetry (ITC) experiments is limited by the heat produced simply by the injection of ligand into the buffer. Data are thus consistent with the notion that the binding affinity correlates with the onset of chemotaxis, as observed previously for PctA and PctB (51).

**TABLE 1:** Thermodynamic parameters for the titration of P. aeruginosa PctD-LBD and *P. atrosepticum* PacA-LBD with different ligands^*a*^

Protein	Compound	*K_D_* (μM)	Δ*H* (kcal/mol)
PctD(PA4633)-LBD	Acetylcholine	23 ± 1	−12.2 ± 1.0
PctD(PA4633)-LBD	Choline	2.6 ± 0.1	−20.3 ± 0.3
PctD(PA4633)-LBD	Betaine	990 ± 59	−2.4 ± 4.1
PctD(PA4633)-LBD	l-carnitine	No binding	
PacA(ECA_RS10935)-LBD	Acetylcholine	No binding	
PacA(ECA_RS10935)-LBD	Choline	113 ± 16.4	−2.1 ± 0.2
PacA(ECA_RS10935)-LBD	Betaine	7.5 ± 0.1	−16.1 ± 0.1
PacA(ECA_RS10935)-LBD	l-carnitine	20 ± 2	−4.9 ± 0.4

a The corresponding titration data are shown in Fig. 2 and 6, respectively.

**FIG 3:**
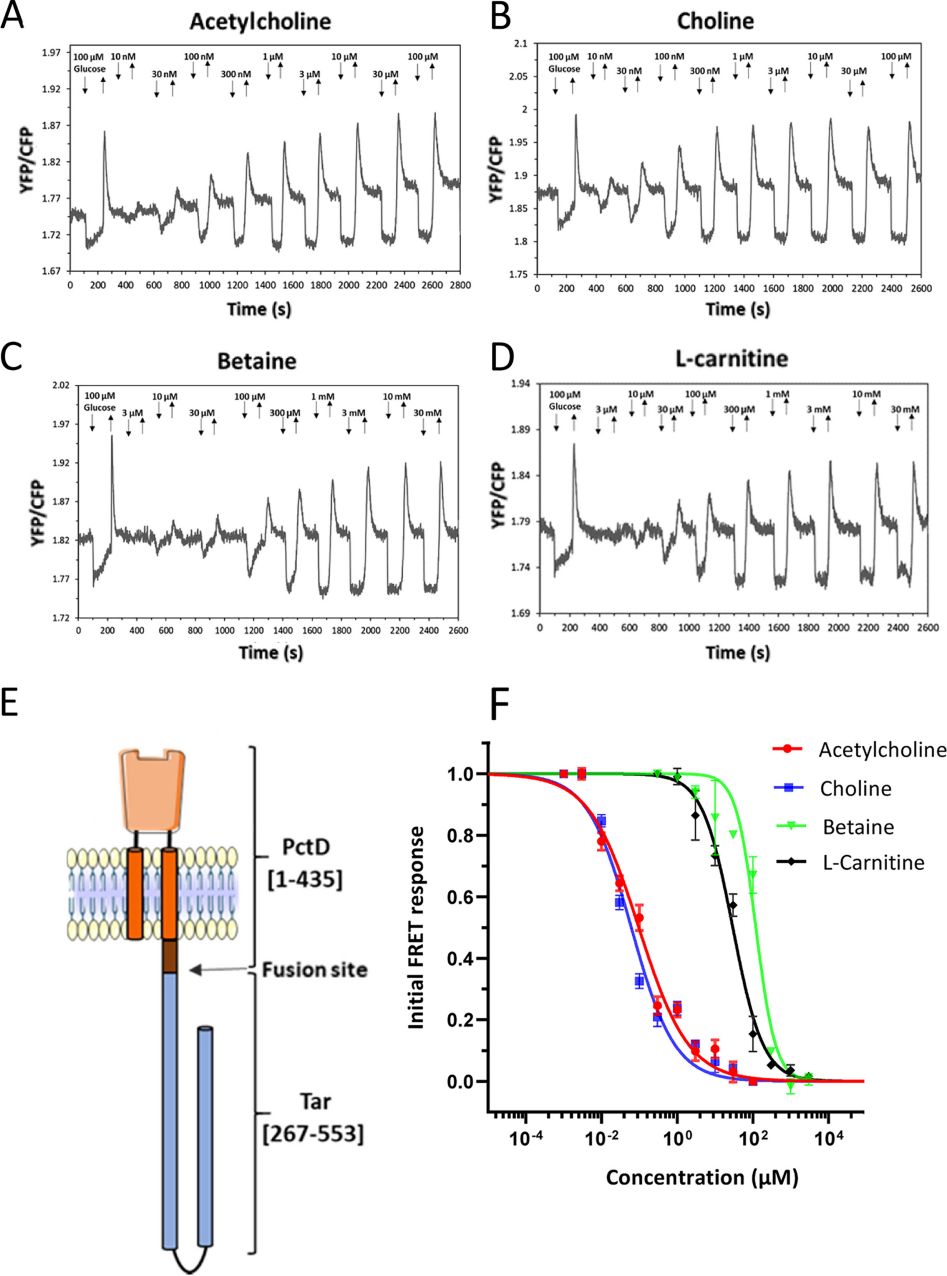
Characterization of responses mediated by a PctD-Tar hybrid using FRET measurements in E. coli. (A to D) FRET responses of buffer-adapted E. coli cells expressing PctD-Tar as the sole receptor upon stepwise addition (down arrow) and subsequent removal (up arrow) of the indicated concentrations of acetylcholine (A), choline (B), betaine (C), and l-carnitine (D). (E) Cartoon representation of the hybrid PctD-Tar. (F) Corresponding dose-response curves of responses mediated by PctD-Tar. The amplitudes of the initial FRET responses were calculated from changes in the ratio of YFP/CFP fluorescence after stimulation with the indicated ligand concentrations and normalized to the saturated response. Error bars indicate the standard errors of three independent experiments; wherever invisible, error bars are smaller than the symbol size. Data were fitted using the Hill equation, with the EC_50_ (half-maximal effective concentration) fit values being 0.07 ± 0.02 μM for acetylcholine, 0.04 ± 0.01 μM for choline, 150 ± 25 μM for betaine, and 31 ± 6 μM for l-carnitine.

In order to confirm that binding of these quaternary amines mediates the chemotaxis response, we constructed a chimeric receptor by fusing the PctD-LBD (including transmembrane helices and HAMP domain) to the signaling domain of the E. coli chemoreceptor Tar (Fig. 3E). Similar chimeras were previously used to investigate chemotaxis signaling induced by binding of chemoeffectors to the LBDs of PctA, PctB, and PctC by measuring the responses that these hybrid receptors mediate in E. coli using Förster resonance energy transfer (FRET) (9, 51). These FRET measurements rely on stimulation-dependent interaction between the chemotaxis response regulator CheY fused to yellow fluorescent protein (CheY-YFP) and its phosphatase CheZ fused to cyan fluorescent protein (CheZ-CFP) (52). The interaction between CheY and CheZ depends on the phosphorylation state of CheY. FRET measurements serve as a precise readout of pathway activity and were found to correlate linearly with physiological responses (53, 54). When PctD-Tar was expressed as the sole chemoreceptor in the E. coli FRET strain VS181/pVS88, clear attractant responses were observed in the submicromolar concentration range for acetylcholine and choline (Fig. 3A and B) and in the micromolar concentration range for betaine and l-carnitine (Fig. 3C and D). The obtained half-maximal effective concentration (EC_50_) values (Fig. 3F) were generally consistent with the relative potency of these ligands as chemoeffectors for P. aeruginosa (Fig. 1) and with the ITC results (Fig. 2) when the signal amplification by the E. coli chemotaxis system is taken into account (52). No responses to these quaternary amines were observed with the E. coli FRET strain expressing only full-length Tar (Fig. S4), confirming the specificity of signaling via the sensory domain of PctD. Choline and acetylcholine also elicited much stronger chemoattraction when the receptorless E. coli strain UU1250 (55) expressing PctD-Tar was tested in a microfluidic assay (Fig. S5).

10.1128/mbio.03458-21.4 FIG S4 FRET measurements of the Tar response to PctD chemoeffectors. Measurements were performed for receptorless E. coli cells expressing wild-type Tar as the sole receptor along with the FRET reporter plasmid. (A to D) Cells were stimulated by the stepwise addition (down arrow) and subsequent removal (up arrow) of the indicated concentrations of acetylcholine (A), choline (B), betaine (C), and l-carnitine (D) in a flow chamber. Download FIG S4, JPG file, 0.1 MB. Copyright © 2022 Matilla et al. 2022 Matilla et al. https://creativecommons.org/licenses/by/4.0/ This content is distributed under the terms of the Creative Commons Attribution 4.0 International license.

10.1128/mbio.03458-21.5 FIG S5 Microfluidic assay of the chemotactic response of E. coli strain UU1250 expressing PctD-Tar as the sole receptor. Relative cell density (fluorescence intensity) in the observation channel over time in gradients of acetylcholine, choline, betaine, or l-carnitine with 50 mM in the source channel as indicated, or in a control channel with tethering buffer. Cell density in the observation channel before ligand stimulation (*t* = 0) was normalized to be 1 in each case. Error bars indicate the standard deviation for three independent biological replicates. Download FIG S5, JPG file, 0.1 MB. Copyright © 2022 Matilla et al. 2022 Matilla et al. https://creativecommons.org/licenses/by/4.0/ This content is distributed under the terms of the Creative Commons Attribution 4.0 International license.

### The three-dimensional structure of PctD-LBD in complex with acetylcholine and choline.

**FIG 4:**
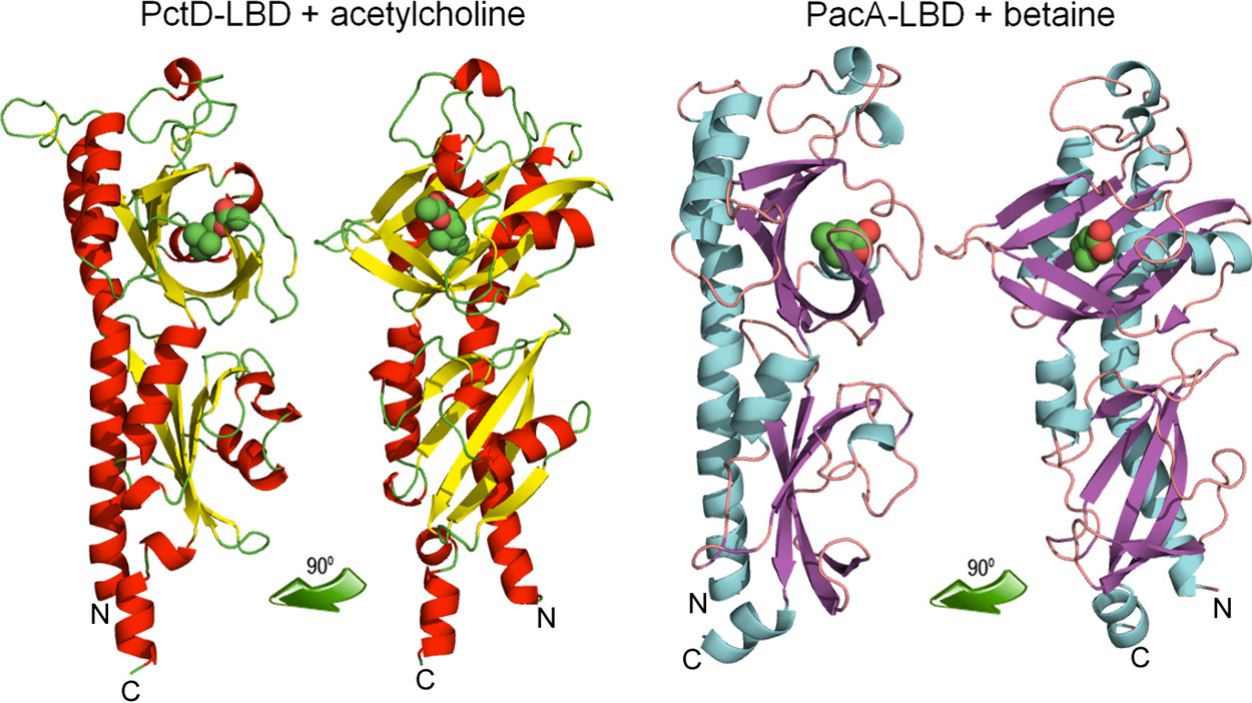
The three-dimensional structures of the ligand-binding domains of the PctD chemoreceptor of P. aeruginosa PAO1 in complex with acetylcholine and the PacA chemoreceptor of *P. atrosepticum* SCRI1043 in complex with betaine. Structures are shown in two different orientations. Bound ligands are shown in space-filling mode.

To understand in molecular detail the mechanism of ligand recognition by PctD, we carried out crystallization trials of PctD-LBD in its ligand-free state and in complex with the identified ligands. Crystals formed in the presence of acetylcholine and choline, and atomic structures were determined and refined to a resolution of 1.8 and 2.0 Å, respectively. The structures showed the typical dCache fold (Fig. 4), and alignments with all structures present in the protein data bank (Table S1) showed that it is most similar to the LBD of the TlpQ chemoreceptor from P. aeruginosa in complex with histamine (10), another neurotransmitter.

10.1128/mbio.03458-21.8 TABLE S1 Results from structural alignments of the PctD-LBD structure (in complex with acetylcholine) and the PacA-LBD structure (in complex with betaine) with structures deposited in the Protein Data Bank*^a^*.^*a*^Shown are the top 15 structures according to the Z-score. Alignments were made with the DALI server (108). Download Table S1, DOCX file, 0.03 MB. Copyright © 2022 Matilla et al. 2022 Matilla et al. https://creativecommons.org/licenses/by/4.0/ This content is distributed under the terms of the Creative Commons Attribution 4.0 International license.

**FIG 5:**
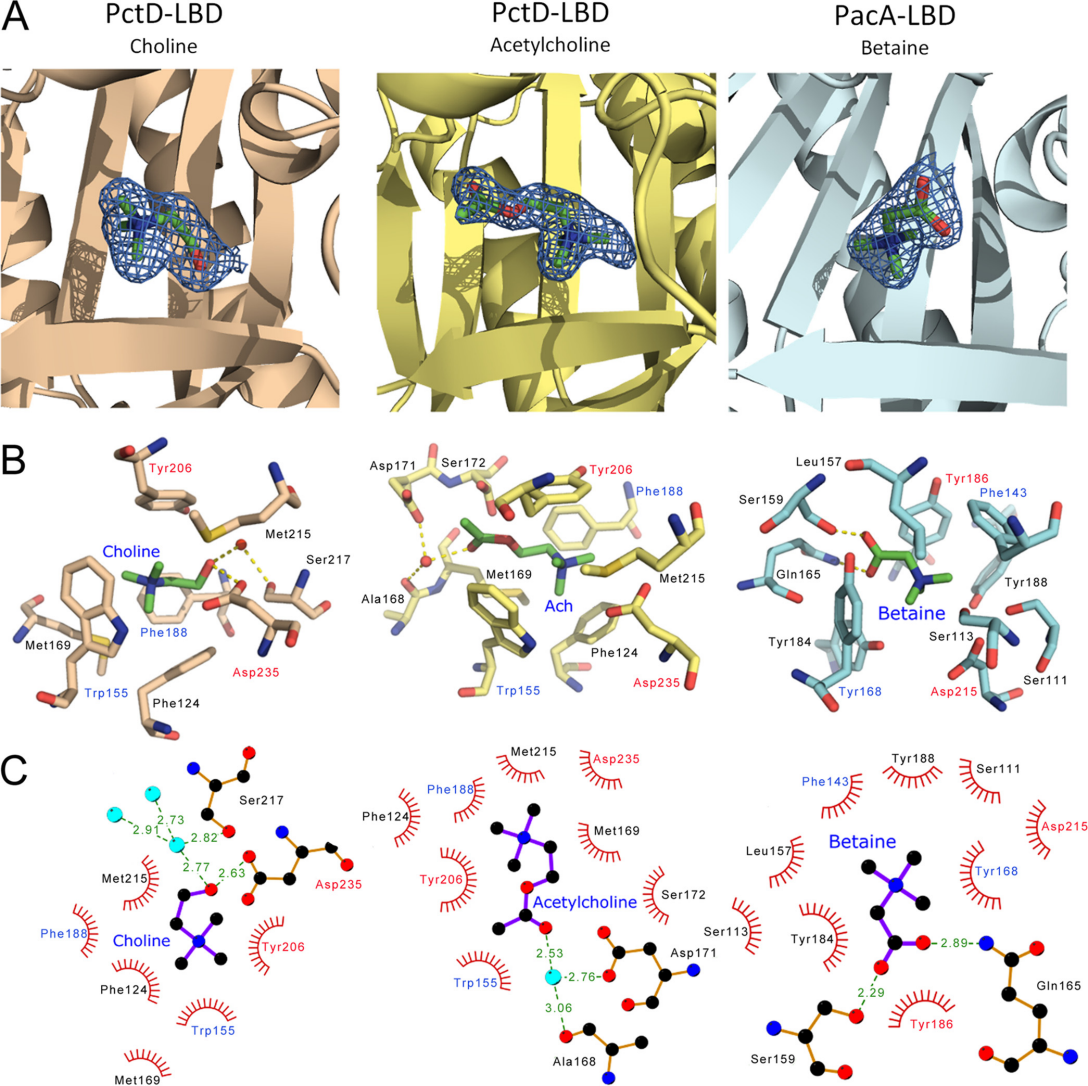
The molecular detail of signal recognition by the PctD and PacA chemoreceptors. (A) A zoom view of the ligand-binding sites. The mesh representation of the final |2Fo–Fc| electron density map is contoured at approximately 1.0 σ. Ligands are shown in stick mode. (B) Amino acids involved in ligand binding. Hydrogen bonds are shown as dotted lines. Amino acids that are conserved in the ligand-binding pockets of PctD and PacA are labeled in red (fully conserved) and blue (aromatic amino acid conserved). Ach, acetylcholine. (C) Schematic view of ligand binding as generated by LigPlot software (106). Hydrophobic interactions are shown as spoked arcs, and hydrogen bonds as dashed green lines (Distances (in Å) are indicated). Cyan spheres are water molecules. Amino acids that are conserved in the ligand-binding pockets of PctD and PacA are labeled in red (fully conserved) and blue (aromatic amino acid conserved).

Among other similar structures are the McpX chemoreceptor for quaternary amines (56), several amino acid sensing chemoreceptors (57, 58), and the DctB and KinD sensor kinases (59, 60) that bind organic acids. Well-defined electron density was observed for choline and acetylcholine, which permitted the precise placement of ligand structures (Fig. 5A). As in the majority of dCache domains, the ligand was bound to the membrane-distal module (Fig. 4). The quaternary amine moiety of both ligands is coordinated by hydrophobic interactions with a number of aromatic amino acids (Phe124, Phe188, Tyr206, and Trp155) and two methionine residues (Met169 and Met215). However, the tails of the two ligands point in opposing directions (Fig. 5). The choline tail interacts with Ser217 and Asp235, whereas the acetylcholine tail interacts with Ala168, Asp171, and Ser172 (Fig. 5).

### Identification of PacA from Pectobacterium atrosepticum that binds quaternary amines but not acetylcholine.

We wanted to advance our understanding of the molecular mechanism for acetylcholine binding at dCache domains. Pectobacterium atrosepticum SCRI1043 is used in our laboratory to investigate chemotaxis of plant pathogens. This strain showed low but reproducible chemotaxis to betaine, choline, and l-carnitine, but it failed to respond to acetylcholine even at 1 mM (Fig. S6). Of the 36 chemoreceptors of SCRI1043, two (ECA_RS10935 and ECA_RS05475) possess a dCache type LBD (61). The LBDs of these receptors share only 20% and 17% sequence identity with PctD, respectively (Fig. S2). We hypothesized that one of these receptors is responsible for the responses to quaternary amines.

10.1128/mbio.03458-21.6 FIG S6 Capillary chemotaxis assays of *P. atrosepticum* SCRI1043 to 1 mM choline, betaine, l-carnitine, and acetylcholine. Data were corrected with the number of cells that swam into buffer-containing capillaries (3,424 ± 417). Data are the means and standard deviations from triplicate analysis of three biological replicates. Download FIG S6, JPG file, 0.1 MB. Copyright © 2022 Matilla et al. 2022 Matilla et al. https://creativecommons.org/licenses/by/4.0/ This content is distributed under the terms of the Creative Commons Attribution 4.0 International license.

**FIG 6:**
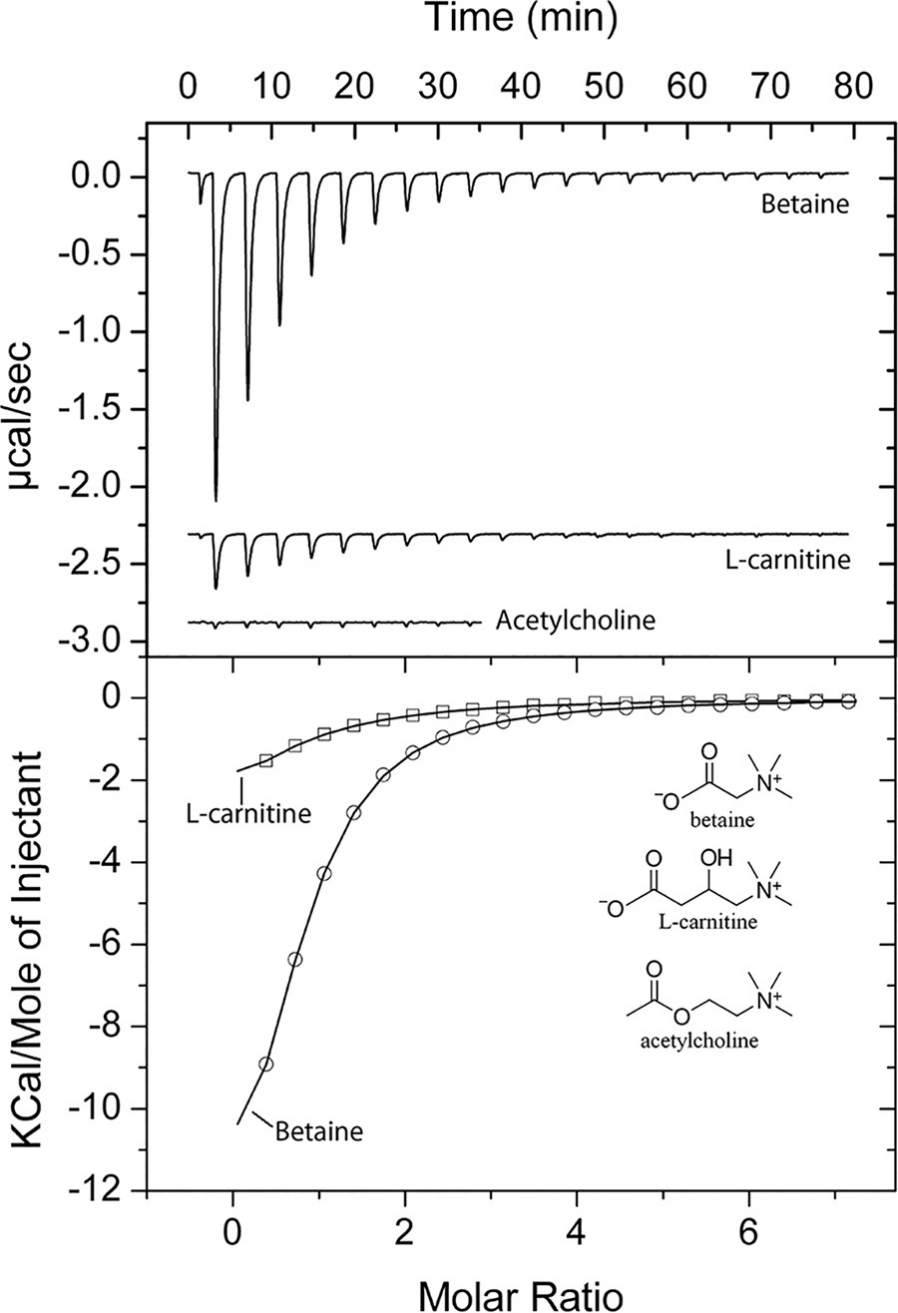
Isothermal titration calorimetry study of the binding of different ligands to the LBD of the *P. atrosepticum* SCRI1043 chemoreceptor ECA_RS10935 (PacA). (Upper panel) Raw data for the titration of 20 μM protein with 9.6-μL aliquots of 1 mM (betaine, carnitine) and 5 mM (acetylcholine) ligand solutions. (Lower panel) Concentration-normalized and dilution heat-corrected raw data for the titration with acetylcholine and choline. The continuous line is the best fit with the “one binding site model” of the MicroCal version of ORIGIN.

To identify the corresponding chemoreceptor, we overexpressed and purified the LBD of ECA_RS10935. Microcalorimetric titrations of the protein with 1 mM solutions of betaine, choline, and l-carnitine showed binding in each case (Fig. 6, Table 1). Betaine bound most tightly (*K_D_* = 7.5 μM), followed by l-carnitine and choline with *K_D_* values of 20 μM and 113 μM, respectively. Importantly, titration with 5 mM acetylcholine (Fig. 6) did not produce any change in heat, indicating an absence of binding.

Given these results, we hypothesized that the observed weak chemotaxis of SCRI1043 to ligands bound by ECA_RS10935 (Fig. S6) may be due to a low level of receptor expression under the experimental conditions. To evaluate this, we constitutively expressed the *ECA_RS10935* gene from a pBBR1MCS-based multicopy vector. *P. atrosepticum* SCRI1043 harboring the resulting plasmid, pBBR_ECA_RS10935, exhibited up to an 8-fold increase in chemotaxis to the corresponding ligands compared to the strain harboring the empty plasmid (Fig. S7). The receptor was thus renamed PacA (Pectobacterium atrosepticum chemoreceptor A).

10.1128/mbio.03458-21.7 FIG S7 Capillary chemotaxis assays of *P. atrosepticum* SCRI1043 containing the empty plasmid pBBR1MCS-2_START and its derivative pBBR_ECA_RS10935 causing expression of chemoreceptor ECA_RS10935 (PacA). Data have been corrected with the number of cells that swam into buffer-containing capillaries (530 ± 233 cells per capillary). Download FIG S7, JPG file, 0.2 MB. Copyright © 2022 Matilla et al. 2022 Matilla et al. https://creativecommons.org/licenses/by/4.0/ This content is distributed under the terms of the Creative Commons Attribution 4.0 International license.

### Comparing the atomic structures of ligand-bound PacA-LBD and PctD-LBD.

We crystalized the PacA-LBD and solved its 3D structure in complex with betaine to a resolution of 1.9 Å (Fig. 4B). The overall structure is highly similar to that of PctD-LBD, with a root mean square deviation of 1.6 Å for the Cα atoms in a structural alignment. This similarity is also evidenced by the fact that the structural homologs of PctD-LBD correspond largely to those of PacA-LBD (Table S1). A well-defined electron density was observed for betaine bound to PacA-LBD (Fig. 5A). The orientation of betaine corresponded to that of acetylcholine in PctD-LBD (Fig. 5B), which was the opposite of the orientation of choline. Comparing the composition of the ligand-binding sites of PctD and PacA resulted in the identification of two conserved amino acids (Tyr and Asp, shown in red in Fig. 5B, C) and two positions occupied by aromatic amino acids in both proteins (shown in blue in Fig. 5B, C). The coordination of the quaternary amine moiety in PacA-LBD is very similar to that of PctD-LBD and primarily involves a number of aromatic amino acids. However, the amino acids that coordinate the acetylcholine tail in PctD-LBD (Ala168, Met169, Asp171, and Ser172) are not conserved in PacA-LBD (Fig. 5B, C, Fig. S2C). These four amino acids in PctD-LBD may thus correspond to a feature of acetylcholine-binding dCache domains.

### Acetylcholine chemotaxis in other species.

**FIG 7:**
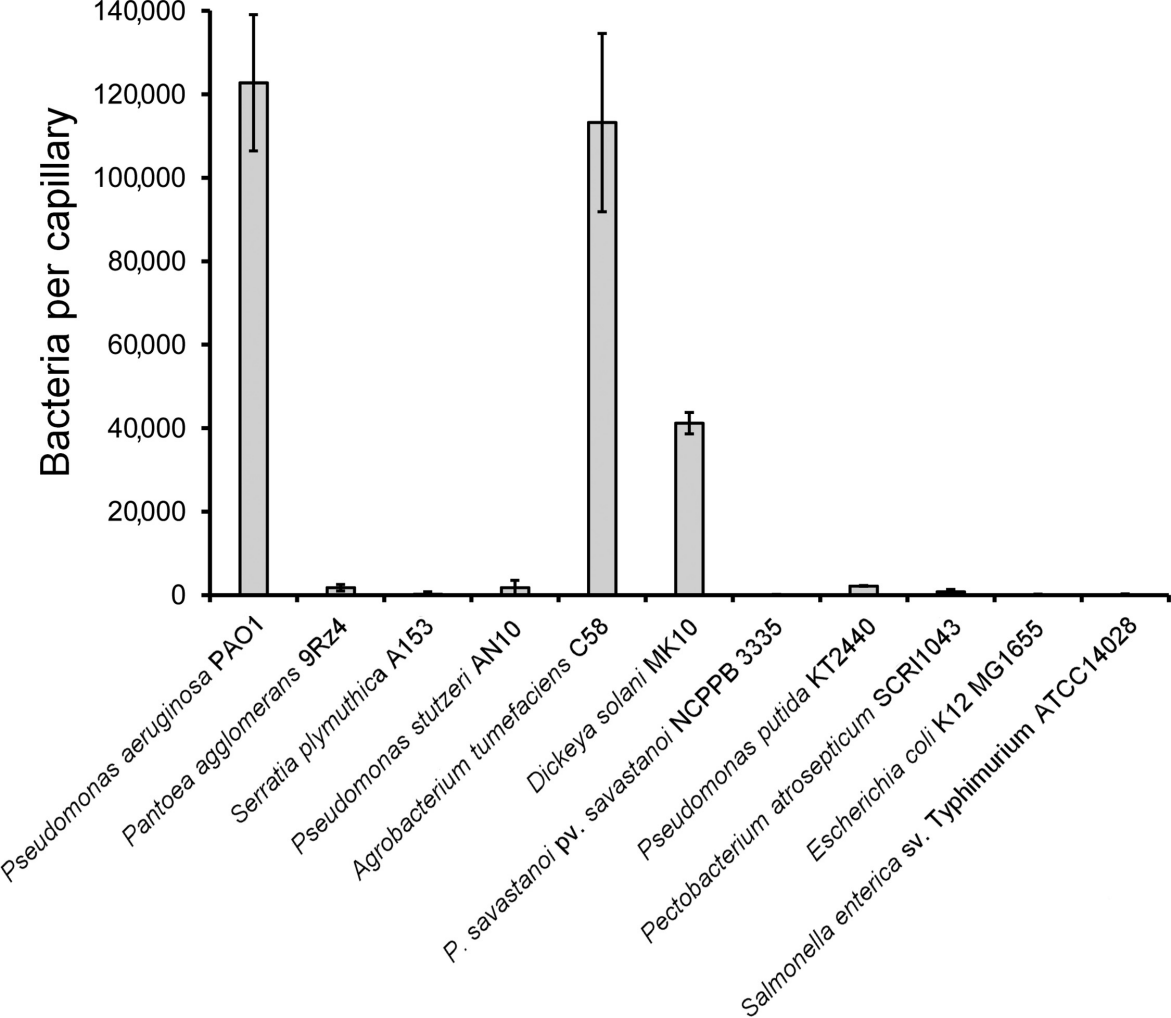
Chemotaxis to acetylcholine in different bacterial species. The results of quantitative capillary chemotaxis assays toward 1 mM acetylcholine are shown. Data are shown as the means and standard deviations of results from three biological replicates conducted in triplicate.

Having obtained the first evidence for bacterial chemotaxis to acetylcholine, we investigated whether other strains show a similar behavior. To this end, we conducted capillary chemotaxis assays to 1 mM acetylcholine using a variety of strains with different lifestyles, including human pathogens, nonpathogenic plant-associated bacteria, soil bacteria, and plant pathogens. Two strains, Agrobacterium tumefaciens C58 and Dickeya solani MK10 showed acetylcholine chemotaxis (Fig. 7). The magnitude in A. tumefaciens was comparable to that in P. aeruginosa, whereas that of *D. solani* was about a third of that observed in P. aeruginosa. Both strains are plant pathogens, indicating that bacteria with different lifestyles inhabiting dissimilar ecological niches are able to perform acetylcholine chemotaxis.

## DISCUSSION

Acetylcholine is one of the key signaling molecules of life. It is best known as the primary neurotransmitter in the vertebrate peripheral nervous system, where it mediates signal transmission at the neuromuscular junction (62). In addition, many human tissues contain nonneuronal acetylcholine, mostly in epithelial cells (airways, alimentary tract, urogenital tract, epidermis), muscles and other mesothelial cells (pleura, pericardium), and endothelial and immune cells (63, 64). The signaling function of nonneuronal acetylcholine modulates diverse processes, including immune and inflammatory responses, wound healing, and development of cancer, cardiovascular, respiratory, digestive, and orthopedic diseases (65, 66). Importantly, nonneuronal acetylcholine is also produced by bacteria, archaea, algae, protozoa, tubellariae, and plants, suggesting an extremely early appearance of acetylcholine in evolution (63). Acetylcholine mediates interkingdom and interbacterial communication (67). It has, for example, been identified as a key regulator of the interaction between microbes and the human immune system (68).

The acetylcholine-mediated interdomain communication between bacteria and other kingdoms is bi-directional, and there are several reports indicating that bacteria perceive acetylcholine. For example, acetylcholine is not a chemoeffector for E. coli, but it inhibits chemotaxis to aspartate (69). In another study, acetylcholine was found to reduce chemotaxis of Pseudomonas fluorescens to l-Leu (70). However, the mode by which acetylcholine is perceived is unknown. In Bacillus subtilis, the transcriptional regulator BmrR binds acetylcholine with significant affinity (*K_D_* = 6.6 μM) to control the expression of the Bmr multidrug efflux pump (71). In S. meliloti, the solute-binding protein ChoX binds acetylcholine (72). In Dickeya dadantii, acetylcholine was identified as a competitive antagonist that interacts with a ligand-gated ion channel (73). To the best of our knowledge, our data constitute the first report that bacteria sense acetylcholine as a strong chemoattractant via chemoreceptors. Because acetylcholine is a crucial signal molecule, it is reasonable to suggest that chemotaxis to acetylcholine promotes the virulence of P. aeruginosa.

Signal transduction consists of converting a signaling input into an output. Here, we have used ITC to quantify the signaling input (Table 1) and three different approaches to quantify the PctD-mediated signaling output, namely, (i) P. aeruginosa chemotaxis assays (Fig. 1 and Fig. S3), (ii) analysis of E. coli cells harboring a PctD-Tar chimera by FRET (Fig. 3), and (iii) microfluidics measurements (Fig. S5). There was satisfactory agreement between these four data sets. Choline and acetylcholine showed high-affinity binding, with *K_D_* values of 2.6 and 23 μM, respectively. It was shown that the *K_D_* values of 60% of all characterized chemoreceptors are between 1 to 50 μM (4), indicating that the recognition of both chemoeffectors by PctD is in the same range as most that of chemoreceptors. In contrast, betaine bound with low affinity, and L-carnitine binding could not be visualized by ITC (Table 1). In agreement with these data, all three approaches to monitor the signaling output showed highly sensitive responses to choline and acetylcholine, whereas low-sensitivity responses were observed for betaine and L-carnitine. The EC_50_ values derived from FRET experiments (legend to Fig. 3) were well below the *K_D_* values from the ITC binding studies (Table 1), which is an observation that has been made for other chemoreceptors (9, 51) and is likely due to signal amplification in chemoreceptor arrays. It can thus be concluded that the magnitude of signaling input determines the magnitude of signaling output at PctD.

The threshold concentration of acetylcholine for chemotaxis was found to be 1 μM, with a maximal response at 10 μM. How do these parameters compare to physiological acetylcholine concentrations? P. aeruginosa is the primary etiological agent of ulcerative keratitis (74). Acetylcholine concentrations of 100 to 150 μM were detected within the corneal epithelium (75), concentrations comparable to those at which strong chemoattraction is observed (Fig. 1). In many cases, chemotaxis is required for the initial steps of infection, in which sites that are suitable for establishing infection are recognized and colonized. Once infection is established, chemotaxis is no longer essential, and transcription of chemotaxis and motility genes is downregulated (76). A possible involvement of the PctD chemoreceptor in virulence is supported by the downregulation of *pctD* transcript levels in human sputum (77, 78), human burn wound infections (78), and mouse lung infection (79) compared to the levels seen during *in vitro* growth.

Experiments are needed to establish whether other bacteria also possess acetylcholine receptors. Overall sequence similarity with LBDs of known ligand profile does not permit prediction of the ligand recognized. However, comparison of the three-dimensional structures of ligand-bound PctD-LBD and PacA-LBD identified the amino acids uniquely involved in acetylcholine recognition. To identify potential acetylcholine-binding receptors in other species, precise models of dCache domains can be generated using AlphaFold (80) and inspected for the presence of these amino acids.

**FIG 8:**
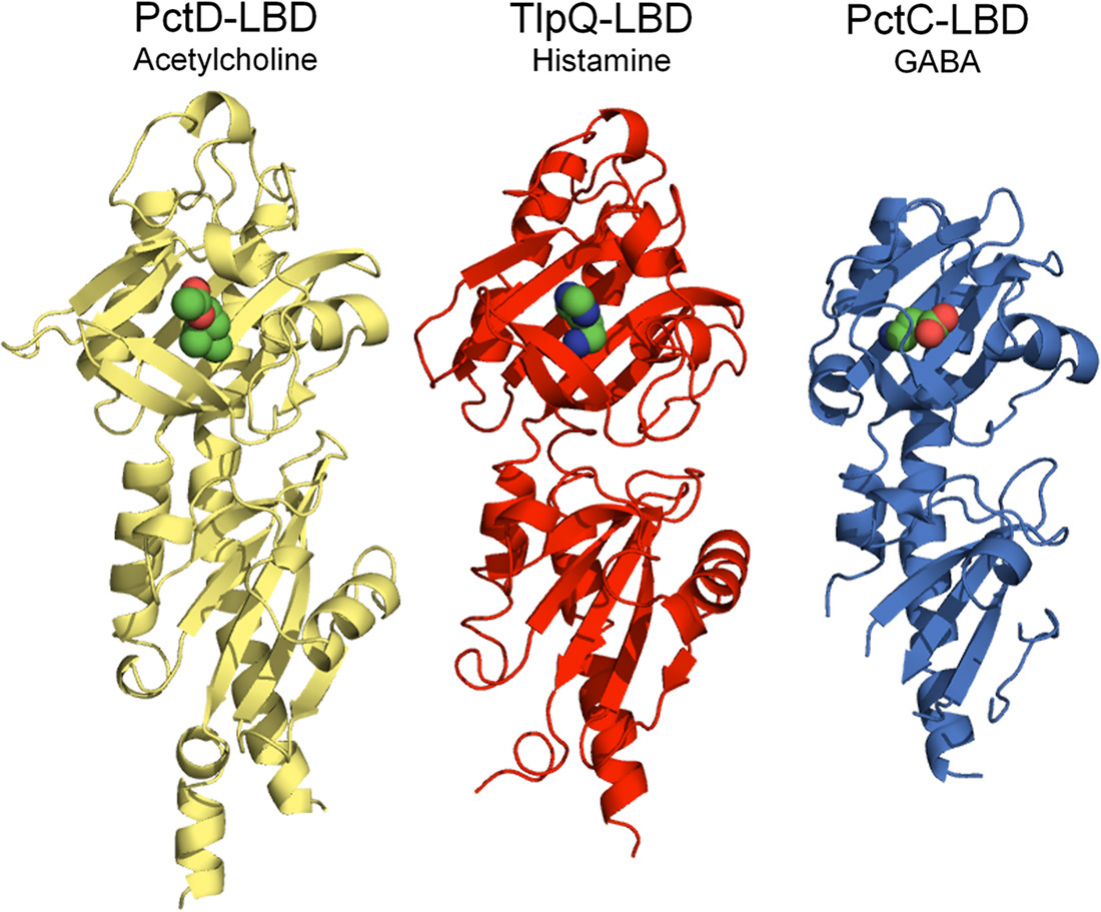
The three dCache domain-containing chemoreceptors of P. aeruginosa PAO1 that mediate chemotaxis to important neurotransmitters. The 3D structures of the ligand-binding domains of PctD in complex with acetylcholine (PDB ID 7PRR), TlpQ in complex with histamine (PDB ID 6FU4) (10), and PctC in complex with GABA (PDB ID 5LTV) (37) are shown. The respective *K_D_* values are 23 μM, 1.2 μM, and 0.64 μM (10, 39).

PctD was determined to bind acetylcholine, a central neurotransmitter and signal molecule. Interestingly, two PctD orthologs in P. aeruginosa, PctC and TlpQ, bind and mediate chemoattraction to other neurotransmitters. GABA is the preferred ligand for the PctC chemoreceptor (*K_D_* = 1.2 μM) (39), and histamine binds TlpQ with a *K_D_* of 0.64 μM. P. aeruginosa exhibits attraction to concentrations of histamine as low as 500 nM (10). The three-dimensional structures of the LBDs of these three dCache family receptors complexed with their respective ligands have been solved (Fig. 8). Acetylcholine, GABA, and histamine are human neurotransmitters and signal molecules involved in interkingdom communication. Therefore, the existence of three chemoreceptors for their recognition by P. aeruginosa suggests a particular relevance of chemoattraction to these compounds. Chemoattraction to neurotransmitters has also been observed in E. coli. E. coli is strongly attracted to norepinephrine (5), a response that requires the conversion of norepinephrine to dihydroxymandelic acid, which is then sensed by the LBD of the Tsr chemoreceptor (5, 81). This chemotaxis response is of physiological relevance, as in enterohemorrhagic E. coli, norepinephrine controls the expression of many virulence genes, a response mediated by the QseC sensor kinase (82–84).

We have tested other bacteria for chemotaxis to acetylcholine. Significant responses were seen with Agrobacterium tumefaciens and Dickeya solani, two plant pathogens (Fig. 7). No chemotaxis to acetylcholine was observed in a number of other plant pathogens and nonpathogenic plant-associated species. Acetylcholine has been detected in many plant taxons and is considered to be a plant hormone (85). Within the plant, it is ubiquitously distributed, ranging from seeds and cotyledons to roots, shoots, and leaves (86). Acetylcholine regulates vital plant activities such as seed germination and plant growth. It mimics the action of red light, influencing leaf movement and membrane permeability to ions and modifying enzyme activities and metabolic processes (85). Acetylcholine concentrations in plants are typically in the lower μM range but can be as high as 100 μM (86, 87). Future investigations will be necessary to establish the physiological relevance of chemotaxis to acetylcholine in plant pathogens. Our study lays the foundation for studies to assess the phylogenetic spread of acetylcholine chemotaxis and to assess its physiological relevance in bacteria.

## MATERIALS AND METHODS

### Strains, plasmids, and culture conditions.

The bacterial strains, plasmids, and oligonucleotides used are listed in Table S2. E. coli strains were grown in LB medium at 37°C. E. coli DH5α was used as a host for gene cloning. When necessary, antibiotics were used at the following concentrations: kanamycin, 25 μg/mL (E. coli strains); ampicillin, 100 μg/mL (E. coli strains); chloramphenicol, 17 μg/mL (E. coli strains); tetracycline, 50 μg/mL (P. aeruginosa strains).

10.1128/mbio.03458-21.9 TABLE S2 Strains, plasmids, and oligonucleotides used in this study. Download Table S2, DOCX file, 0.1 MB. Copyright © 2022 Matilla et al. 2022 Matilla et al. https://creativecommons.org/licenses/by/4.0/ This content is distributed under the terms of the Creative Commons Attribution 4.0 International license.

### Construction of plasmids.

The DNA fragment encoding the LBD of P. aeruginosa PAO1 chemoreceptor PA4633 (amino acids 32 to 361) was amplified by PCR from genomic DNA and cloned into the NdeI and XhoI sites of pET28b(+) to generate plasmid pET28_PA4633-LBD. The DNA fragment encoding the LBD of *P. atrosepticum* SCRI1043 chemoreceptor ECA_RS10935 (amino acids 37 to 317) was amplified by PCR from genomic DNA. The resulting PCR fragment was then submitted to restriction free-cloning into pET28b(+) as described in reference 88. For the construction of the plasmid for complementation assays, the *pa4633* gene was PCR amplified and cloned into pBBR1MCS-2_START. The resulting plasmid was transformed into P. aeruginosa PAO1-PA4633 by electroporation. The DNA sequence of gene *ECA_RS10935* was amplified from the genomic DNA of *P. atrosepticum* SCRI1043 and cloned into plasmid pBBR1MCS-2_START digested with NdeI and BamHI, resulting in pBBR_ECA_RS10935. The hybrid gene encoding PctD-Tar was constructed using PCR and inserted under the salicylate-inducible promoter into pKG116 using NdeI and BamHI sites to yield plasmid pVS1743. All plasmids were verified by sequencing the inserts and flanking regions.

### Protein overexpression and purification.

E. coli BL21-AI and E. coli BL21(DE3) harboring plasmids pET28_PA4633-LBD and pET28_ECA_RS10935-LBD, respectively, were grown in 2-L Erlenmeyer flasks containing 500 mL LB medium supplemented with kanamycin. Cultures were grown under continuous stirring (200 rpm) at 30°C. At an optical density at 660 nm (OD_660_) of 0.5, PA4633-LBD expression was induced by the addition of 0.2% (wt/vol) l-arabinose and 1 mM isopropyl-β-d-thiogalactopyranoside (IPTG). Growth was continued at 30°C for 5 h, and cells were harvested by centrifugation at 20,000 × *g* for 20 min at 4°C. ECA_RS10935-LBD expression was induced by adding 0.1 mM IPTG at an OD_660_ of 0.5, and growth was continued overnight at 18°C, prior to cell harvest by centrifugation at 20,000 × *g* for 20 min. Proteins were purified by metal affinity chromatography. Briefly, the cell pellets of PA4633-LBD and ECA_RS10935-LBD were resuspended in buffer A (30 mM Tris/HCl, 300 mM NaCl, 10 mM imidazole, 5% [vol/vol] glycerol, pH 8.0) or buffer B (40 mM KH_2_PO_4_*/*K_2_HPO_4_, 10 mM imidazole, 10% [vol/vol] glycerol, pH 7.0), respectively, containing cOmplete protease inhibitor (Roche) and Benzonase (Merck). Cells were then broken by French press treatment at a gauge pressure of 62.5 lb/in^2^. After centrifugation at 10,000 × *g* for 1 h, the supernatant was loaded onto a 5-mL HisTrap column (Amersham Bioscience) equilibrated with buffers A or B. Proteins were eluted by a gradient of 40 to 500 mM imidazole in the same buffers.

### Isothermal titration calorimetry.

Measurements were made using a VP-ITC microcalorimeter (MicroCal, Inc., Northampton, MA) at 25°C. PA4633-LBD was dialyzed into 5 mM Tris, 5 mM PIPES [piperazine-N,N′-bis(2-ethanesulfonic acid)], 5 mM MES (morpholineethanesulfonic acid), 150 mM NaCl, 10% (vol/vol) glycerol, pH 7.5, whereas ECA_RS10935-LBD was dialyzed into 40 mM KH_2_PO_4_*/*K_2_HPO_4_, 10% (vol/vol) glycerol, pH 7.0. Proteins at 15 to 22 μM were placed into the sample cell and titrated with 9.6-μL aliquots of 0.5 to 10 mM ligand solutions made up in dialysis buffer. In the absence of binding, the experiment was repeated at 15°C. The mean enthalpies from the injection of ligands into the buffer were subtracted from raw data prior to data fitting using the “one binding site model” of the MicroCal version of the ORIGIN software.

### Chemotaxis assays.

Overnight cultures in M9 minimal medium supplemented with 6 mg/L Fe-citrate, trace elements (89), and 15 mM glucose were used to inoculate fresh medium to an OD_660_ of 0.05. Cells were cultured at 30°C (Pectobacterium atrosepticum, Pantoea agglomerans, Serratia plymuthica, Pseudomonas stutzeri, Agrobacterium tumefaciens, Dickeya solani, Pseudomonas savastanoi pv. *savastanoi*) or 37°C (P. aeruginosa, E. coli, Salmonella enterica serovar Typhimurium) to an OD_660_ of 0.4 to 0.5. Subsequently, cells were washed twice by centrifugation (1,667 × *g* for 5 min at room temperature) and resuspension in chemotaxis buffer (50 mM KH_2_PO_4_*/*K_2_HPO_4_, 20 mM EDTA, 0.05% [vol/vol] glycerol, pH 7.0) and then resuspended in the same buffer at an OD_660_ of 0.1. Aliquots (230 μL) of the resulting cell suspension were placed into the wells of 96-well microtiter plates. Then, 1-μL capillaries (Microcaps, Drummond Scientific) were heat-sealed at one end and filled with buffer (control) or chemoeffector solution prepared in chemotaxis buffer. The capillaries were rinsed with sterile water and immersed into the bacterial suspensions at their open ends. After 30 min, capillaries were removed from the wells, rinsed with sterile water, and emptied into 1 mL of chemotaxis buffer. Serial dilutions were plated onto M9 minimal medium plates supplemented with 20 mM glucose, incubated at 30°C or 37°C prior to colony counting. Data were corrected with the number of cells that swam into buffer containing capillaries. Data are the means and standard deviations of three biological replicates conducted in triplicate.

### Protein crystallization and structure resolution.

Freshly purified PctD-LBD was dialyzed into 5 mM Tris/HCl, 5 mM MES, 5 mM PIPES, 150 mM NaCl, and 10% (vol/vol) glycerol, pH 7.5, and concentrated to 20 mg/mL using 10-kDa cutoff Centricon concentrators (Amicon). For the cocrystallization experiments, ligands were added to a final concentration of 10 mM and incubated on ice for 30 min. The excess of ligand was removed by rounds of concentration and dilution with the above-described buffer. Protein (20 mg/mL) was submitted to crystal screening using the hanging-drop vapor diffusion and capillary counterdiffusion techniques. Vapor diffusion experiments were set up in 24-well crystallization plates (VDX; Hampton Research) using the Hampton Research Screen I. Droplets were prepared by mixing protein with reservoir solution at a 1:1 ratio and equilibrated over a 500-μL reservoir solution. Counterdiffusion experiments were set up by loading protein into 0.2-mm inner diameter capillaries and equilibrated against an excess of precipitant cocktails prepared *ad hoc* (90). A similar procedure was employed to crystallize PacA-LBD, except that protein was at 35 mg/mL and in 5 mM Tris/HCl, 5 mM PIPES, and 5 mM MES, pH 7.4. The conditions that resulted in crystals are given in Table S3. Crystals were equilibrated in mother solution supplemented with either 15% (vol/vol) glycerol or 20% (vol/vol) polyethylene glycol (PEG) 200, recovered by LithoLoops (Molecular Dimensions), and flash-frozen in liquid nitrogen. Data collection was done at beamlines ID30B, ID23-1, and ID30A-3 of the European Synchrotron Radiation Facility (Grenoble, France) and the Xaloc beamline of the Alba Spanish synchrotron radiation source (Barcelona, Spain). Data were indexed and integrated with XDS (91) and scaled and reduced with AIMLESS (92) of the CCP4 program suite (93). PctD-LBD was phased by molecular replacement with MOLREP (94) using a truncated version of the model predicted by RaptorX (95) that was based on templates with PDB ID 6F9G, 6FU4, and 6PZJ. The molecular replacement solution of PacA-LBD was found by implementing the deep learning-based method AlphaFold within the Rossetta predictor software run in the Robetta server (RoseTTAFold) (96) and top ranked in CAMEO (97). Refinement was initiated with phenix.refine (98) and REFMAC5 (99) of the CCP4 program suite. Manual building, water inspection, and ligand identification were done in Coot (100), and final refinement was assessed, including titration-libration-screw parameterization (101). Models were verified with Molprobity (102) and the PDB validation server prior to deposition at the PDBe (103). The crystallographic data statistics and final model characteristics are provided in Table S3.

10.1128/mbio.03458-21.10 TABLE S3 Crystallization conditions, data collection, and refinement statistics of the PctD-LBD and PacA-LBD three-dimensional structures. Download Table S3, DOCX file, 0.02 MB. Copyright © 2022 Matilla et al. 2022 Matilla et al. https://creativecommons.org/licenses/by/4.0/ This content is distributed under the terms of the Creative Commons Attribution 4.0 International license.

### Growth experiments.

PAO1 was grown overnight in M9 minimal medium containing 10 mM glucose. Cultures were washed twice and then diluted to an OD_600_ of 0.02 in either M9 or M8 medium (M9 minimal medium without NH_4_Cl) containing 10 mM glucose and medium supplemented with each of the compounds present in the Biolog compound array PM2A (https://www.biolog.com/wp-content/uploads/2020/04/00A-042-Rev-C-Phenotype-MicroArrays-1-10-Plate-Maps.pdf) as sole carbon source. Then, 200 μL of these cultures were transferred into microwell plates, and growth at 37°C was followed on a Bioscreen microbiological growth analyzer (Oy Growth Curves Ab Ltd., Helsinki, Finland).

### FRET measurements.

FRET measurements were performed as described previously (52, 54, 104). Cells of chemoreceptorless E. coli strain VS181 expressing PctD-Tar and the CheY-YFP/CheZ-CFP FRET pair were prepared by inoculating 200 μL of the overnight culture into 10 mL tryptone broth (TB; 1% [wt/vol] tryptone and 0.5% [wt/vol] NaCl) supplemented with ampicillin, chloramphenicol, 50 μM IPTG, and 2 μM sodium salicylate and grown in a rotary shaker at 34°C and 275 rpm. Cells were harvested at an OD_600_ of 0.5 by centrifugation, washed with tethering buffer (10 mM KH_2_PO_4_/K_2_HPO_4_, 0.1 mM EDTA, 1 μM methionine, 10 mM sodium lactate, pH 7.0), resuspended in 10 mL tethering buffer, and kept at 4°C. For microscopy, the cells were attached to poly-lysine-coated coverslips for 10 min and mounted into a flow chamber that was maintained under constant flow of 0.3 mL/min of tethering buffer using a syringe pump (Harvard Apparatus) that was also used to add or remove compounds of interest. FRET measurements were performed on an upright fluorescence microscope (Zeiss AxioImager.Z1) equipped with photon counters (Hamamatsu). The fluorescence signals were recorded and analyzed as described previously (52, 54).

### Microfluidic assay.

The microfluidic assay was performed as previously described, using a chip with 24 parallel microchannels (105). In brief, cells of the receptorless E. coli strain UU1250 expressing green fluorescent protein (GFP) and PctD-Tar were grown at 34°C in TB supplemented with ampicillin, chloramphenicol, 100 μM IPTG, and 2 μM sodium salicylate to an OD_600_ of 0.5. Cells were harvested by centrifugation and washed twice with tethering buffer. Chemoeffectors were dissolved in tethering buffer at a concentration of 50 mM, and the pH was adjusted to 7.0. The chemical source microchannels were filled with 4% (wt/vol) agarose gel to create a semipermeable barrier. E. coli cells were added to the reservoir well and allowed to spread for 30 min into the channels. Compounds were added to the source well and allowed to form a concentration gradient. Cell fluorescence was recorded with a Ti-E inverted microscope system (Nikon Instruments Europe BV, Amsterdam, Netherlands) using a 20×lens objective. Data were analyzed using ImageJ (Wayne Rasband, NIH, USA).

### Data availability.

The 3D structures reported have been deposited at the protein data bank with the accession codes 7PRQ, 7PRR, and 7PSG.

## References

[1] Sanchis-Lopez C, Cerna-Vargas JP, Santamaria-Hernando S, Ramos C, Krell T, Rodriguez-Palenzuela P, Lopez-Solanilla E, Huerta-Cepas J, Rodriguez-Herva JJ. 2021. Prevalence and specificity of chemoreceptor profiles in plant-associated bacteria. mSystems 6:e00951-21. doi:10.1128/mSystems.00951-21. PMC8547431 34546073

[2] Parkinson JS, Hazelbauer GL, Falke JJ. 2015. Signaling and sensory adaptation in *Escherichia coli* chemoreceptors: 2015 update. Trends Microbiol 23:257–266. doi:10.1016/j.tim.2015.03.003. 25834953 PMC4417406

[3] Bi S, Sourjik V. 2018. Stimulus sensing and signal processing in bacterial chemotaxis. Curr Opin Microbiol 45:22–29. doi:10.1016/j.mib.2018.02.002. 29459288

[4] Matilla MA, Velando F, Martin-Mora D, Monteagudo-Cascales E, Krell T. 2022. A catalogue of signal molecules that interact with sensor kinases, chemoreceptors and transcriptional regulators. FEMS Microbiol Rev 46:fuab043–4000. doi:10.1093/femsre/fuab043. 34424339

[5] Pasupuleti S, Sule N, Cohn WB, MacKenzie DS, Jayaraman A, Manson MD. 2014. Chemotaxis of *Escherichia coli* to norepinephrine (NE) requires conversion of NE to 3,4-dihydroxymandelic acid. J Bacteriol 196:3992–4000. doi:10.1128/JB.02065-14. 25182492 PMC4248876

[6] Laganenka L, Colin R, Sourjik V. 2016. Chemotaxis towards autoinducer 2 mediates autoaggregation in *Escherichia coli*. Nat Commun 7:12984. doi:10.1038/ncomms12984. 27687245 PMC5056481

[7] Lopes JG, Sourjik V. 2018. Chemotaxis of *Escherichia coli* to major hormones and polyamines present in human gut. ISME J 12:2736–2747. doi:10.1038/s41396-018-0227-5. 29995838 PMC6194112

[8] Antunez-Lamas M, Cabrera E, Lopez-Solanilla E, Solano R, González-Melendi P, Chico JM, Toth I, Birch P, Pritchard L, Prichard L, Liu H, Rodriguez-Palenzuela P. 2009. Bacterial chemoattraction towards jasmonate plays a role in the entry of *Dickeya dadantii* through wounded tissues. Mol Microbiol 74:662–671. doi:10.1111/j.1365-2958.2009.06888.x. 19818025

[9] Reyes-Darias JA, Garcia V, Rico-Jimenez M, Corral-Lugo A, Lesouhaitier O, Juarez-Hernandez D, Yang Y, Bi S, Feuilloley M, Munoz-Rojas J, Sourjik V, Krell T. 2015. Specific gamma-aminobutyrate chemotaxis in *pseudomonads* with different lifestyle. Mol Microbiol 97:488–501. doi:10.1111/mmi.13045. 25921834

[10] Corral-Lugo A, Matilla MA, Martin-Mora D, Silva Jimenez H, Mesa Torres N, Kato J, Hida A, Oku S, Conejero-Muriel M, Gavira JA, Krell T. 2018. High-affinity chemotaxis to histamine mediated by the TlpQ chemoreceptor of the human pathogen *Pseudomonas aeruginosa*. mBio 9:e01894-18. doi:10.1128/mBio.01894-18. 30425146 PMC6234866

[11] Ortega A, Zhulin IB, Krell T. 2017. Sensory repertoire of bacterial chemoreceptors. Microbiol Mol Biol Rev 81:e00033-17. doi:10.1128/MMBR.00033-17. PMC5706747 29070658

[12] Elgamoudi BA, Andrianova EP, Shewell LK, Day CJ, King RM, Taha Rahman H, Hartley-Tassell LE, Zhulin IB, Korolik V. 2021. The *Campylobacter jejuni* chemoreceptor Tlp10 has a bimodal ligand-binding domain and specificity for multiple classes of chemoeffectors. Sci Signal 14:eabc8521. doi:10.1126/scisignal.abc8521. 33402336 PMC8112392

[13] Tajima H, Imada K, Sakuma M, Hattori F, Nara T, Kamo N, Homma M, Kawagishi I. 2011. Ligand specificity determined by differentially arranged common ligand-binding residues in bacterial amino acid chemoreceptors Tsr and Tar. J Biol Chem 286:42200–42210. doi:10.1074/jbc.M111.221887. 21979954 PMC3234949

[14] Milligan DL, Koshland DE, Jr. 1993. Purification and characterization of the periplasmic domain of the aspartate chemoreceptor. J Biol Chem 268:19991–19997. doi:10.1016/S0021-9258(20)80684-X. 8397194

[15] Lin LN, Li J, Brandts JF, Weis RM. 1994. The serine receptor of bacterial chemotaxis exhibits half-site saturation for serine binding. Biochemistry 33:6564–6570. doi:10.1021/bi00187a025. 8204592

[16] Milo R, Jorgensen P, Moran U, Weber G, Springer M. 2010. BioNumbers: the database of key numbers in molecular and cell biology. Nucleic Acids Res 38:D750–D753. doi:10.1093/nar/gkp889. 19854939 PMC2808940

[17] Colin R, Sourjik V. 2017. Emergent properties of bacterial chemotaxis pathway. Curr Opin Microbiol 39:24–33. doi:10.1016/j.mib.2017.07.004. 28822274

[18] Hosking ER, Vogt C, Bakker EP, Manson MD. 2006. The *Escherichia coli* MotAB proton channel unplugged. J Mol Biol 364:921–937. doi:10.1016/j.jmb.2006.09.035. 17052729

[19] Martinez-Garcia E, Nikel PI, Chavarria M, de Lorenzo V. 2014. The metabolic cost of flagellar motion in *Pseudomonas putida* KT2440. Environ Microbiol 16:291–303. doi:10.1111/1462-2920.12309. 24148021

[20] Colin R, Ni B, Laganenka L, Sourjik V. 2021. Multiple functions of flagellar motility and chemotaxis in bacterial physiology. FEMS Microbiol Rev 45:fuab038. doi:10.1093/femsre/fuab038. 34227665 PMC8632791

[21] Lacal J, Garcia-Fontana C, Munoz-Martinez F, Ramos JL, Krell T. 2010. Sensing of environmental signals: classification of chemoreceptors according to the size of their ligand binding regions. Environ Microbiol 12:2873–2884. doi:10.1111/j.1462-2920.2010.02325.x. 20738376

[22] Johnson KS, Ottemann KM. 2018. Colonization, localization, and inflammation: the roles of *H. pylori* chemotaxis *in vivo*. Curr Opin Microbiol 41:51–57. doi:10.1016/j.mib.2017.11.019. 29202336 PMC5862749

[23] Perkins A, Tudorica DA, Amieva MR, Remington SJ, Guillemin K. 2019. *Helicobacter pylori* senses bleach (HOCl) as a chemoattractant using a cytosolic chemoreceptor. PLoS Biol 17:e3000395. doi:10.1371/journal.pbio.3000395. 31465435 PMC6715182

[24] Hanyu H, Engevik KA, Matthis AL, Ottemann KM, Montrose MH, Aihara E. 2019. *Helicobacter pylori* uses the TlpB receptor to sense sites of gastric injury. Infect Immun 87:e00202-19. doi:10.1128/IAI.00202-19. 31262979 PMC6704605

[25] Crone S, Vives-Flórez M, Kvich L, Saunders AM, Malone M, Nicolaisen MH, Martínez-García E, Rojas-Acosta C, Catalina Gomez-Puerto M, Calum H, Whiteley M, Kolter R, Bjarnsholt T. 2020. The environmental occurrence of *Pseudomonas aeruginosa*. APMIS 128:220–231. doi:10.1111/apm.13010. 31709616

[26] Bel Hadj Ahmed A, Salah Abbassi M, Rojo-Bezares B, Ruiz-Roldán L, Dhahri R, Mehri I, Sáenz Y, Hassen A. 2020. Characterization of *Pseudomonas aeruginosa* isolated from various environmental niches: new STs and occurrence of antibiotic susceptible “high-risk clones”. Int J Environ Health Res 30:643–652. doi:10.1080/09603123.2019.1616080. 31094221

[27] Bédard E, Prévost M, Déziel E. 2016. *Pseudomonas aeruginosa* in premise plumbing of large buildings. Microbiologyopen 5:937–956. doi:10.1002/mbo3.391. 27353357 PMC5221438

[28] Iglewski BH. 1996. *Pseudomonas*. *In* Baron S (ed), Medical microbiology, 4th ed. University of Texas Medical Branch at Galveston, Galveston, TX.

[29] Morata L, Cobos-Trigueros N, Martínez JA, Soriano A, Almela M, Marco F, Sterzik H, Núñez R, Hernández C, Mensa J. 2012. Influence of multidrug resistance and appropriate empirical therapy on the 30-day mortality rate of *Pseudomonas aeruginosa* bacteremia. Antimicrob Agents Chemother 56:4833–4837. doi:10.1128/AAC.00750-12. 22751533 PMC3421866

[30] Sindeldecker D, Stoodley P. 2021. The many antibiotic resistance and tolerance strategies of *Pseudomonas aeruginosa*. Biofilm 3:100056. doi:10.1016/j.bioflm.2021.100056. 34471871 PMC8387898

[31] Jean SS, Chang YC, Lin WC, Lee WS, Hsueh PR, Hsu CW. 2020. Epidemiology, treatment, and prevention of nosocomial bacterial pneumonia. J Clin Med 9:275. doi:10.3390/jcm9010275. PMC7019939 31963877

[32] Kang CI, Kim SH, Kim HB, Park SW, Choe YJ, Oh MD, Kim EC, Choe KW. 2003. *Pseudomonas aeruginosa* bacteremia: risk factors for mortality and influence of delayed receipt of effective antimicrobial therapy on clinical outcome. Clin Infect Dis 37:745–751. doi:10.1086/377200. 12955633

[33] Walker TS, Bais HP, Deziel E, Schweizer HP, Rahme LG, Fall R, Vivanco JM. 2004. *Pseudomonas aeruginosa*-plant root interactions. Pathogenicity, biofilm formation, and root exudation. Plant Physiol 134:320–331. doi:10.1104/pp.103.027888. 14701912 PMC316311

[34] Rahme LG, Ausubel FM, Cao H, Drenkard E, Goumnerov BC, Lau GW, Mahajan-Miklos S, Plotnikova J, Tan MW, Tsongalis J, Walendziewicz CL, Tompkins RG. 2000. Plants and animals share functionally common bacterial virulence factors. Proc Natl Acad Sci USA 97:8815–8821. doi:10.1073/pnas.97.16.8815. 10922040 PMC34017

[35] Matilla MA, Martin-Mora D, Gavira JA, Krell T. 2021. *Pseudomonas aeruginosa* as a model to study chemosensory pathway signaling. Microbiol Mol Biol Rev 85:e00151-20. doi:10.1128/MMBR.00151-20. 33441490 PMC7849354

[36] Ortega DR, Fleetwood AD, Krell T, Harwood CS, Jensen GJ, Zhulin IB. 2017. Assigning chemoreceptors to chemosensory pathways in *Pseudomonas aeruginosa*. Proc Natl Acad Sci USA 114:12809–12814. doi:10.1073/pnas.1708842114. 29133402 PMC5715753

[37] Gavira JA, Gumerov VM, Rico-Jimenez M, Petukh M, Upadhyay AA, Ortega A, Matilla MA, Zhulin IB, Krell T. 2020. How bacterial chemoreceptors evolve novel ligand specificities. mBio 11:e03066-19. doi:10.1128/mBio.03066-19. 31964737 PMC6974571

[38] Taguchi K, Fukutomi H, Kuroda A, Kato J, Ohtake H. 1997. Genetic identification of chemotactic transducers for amino acids in *Pseudomonas aeruginosa*. Microbiology 143:3223–3229. doi:10.1099/00221287-143-10-3223. 9353923

[39] Rico-Jimenez M, Munoz-Martinez F, Garcia-Fontana C, Fernandez M, Morel B, Ortega A, Ramos JL, Krell T. 2013. Paralogous chemoreceptors mediate chemotaxis towards protein amino acids and the non-protein amino acid gamma-aminobutyrate (GABA). Mol Microbiol 88:1230–1243. doi:10.1111/mmi.12255. 23650915

[40] Rico-Jimenez M, Reyes-Darias JA, Ortega A, Diez Pena AI, Morel B, Krell T. 2016. Two different mechanisms mediate chemotaxis to inorganic phosphate in *Pseudomonas aeruginosa*. Sci Rep 6:28967. doi:10.1038/srep28967. 27353565 PMC4926252

[41] Wu H, Kato J, Kuroda A, Ikeda T, Takiguchi N, Ohtake H. 2000. Identification and characterization of two chemotactic transducers for inorganic phosphate in *Pseudomonas aeruginosa*. J Bacteriol 182:3400–3404. doi:10.1128/JB.182.12.3400-3404.2000. 10852870 PMC101905

[42] Martin-Mora D, Ortega A, Reyes-Darias JA, García V, López-Farfán D, Matilla MA, Krell T. 2016. Identification of a chemoreceptor in *Pseudomonas aeruginosa* that specifically mediates chemotaxis toward alpha-ketoglutarate. Front Microbiol 7:1937. doi:10.3389/fmicb.2016.01937. 27965656 PMC5126104

[43] Martin-Mora D, Ortega A, Matilla MA, Martinez-Rodriguez S, Gavira JA, Krell T. 2019. The molecular mechanism of nitrate chemotaxis via direct ligand binding to the PilJ domain of McpN. mBio 10:e02334-18. doi:10.1128/mBio.02334-18. 30782655 PMC6381276

[44] Martin-Mora D, Ortega A, Perez-Maldonado FJ, Krell T, Matilla MA. 2018. The activity of the C4-dicarboxylic acid chemoreceptor of *Pseudomonas aeruginosa* is controlled by chemoattractants and antagonists. Sci Rep 8:2102. doi:10.1038/s41598-018-20283-7. 29391435 PMC5795001

[45] Alvarez-Ortega C, Harwood CS. 2007. Identification of a malate chemoreceptor in *Pseudomonas aeruginosa* by screening for chemotaxis defects in an energy taxis-deficient mutant. Appl Environ Microbiol 73:7793–7795. doi:10.1128/AEM.01898-07. 17933940 PMC2168054

[46] Kim HE, Shitashiro M, Kuroda A, Takiguchi N, Ohtake H, Kato J. 2006. Identification and characterization of the chemotactic transducer in *Pseudomonas aeruginosa* PAO1 for positive chemotaxis to trichloroethylene. J Bacteriol 188:6700–6702. doi:10.1128/JB.00584-06. 16952963 PMC1595487

[47] Hong CS, Shitashiro M, Kuroda A, Ikeda T, Takiguchi N, Ohtake H, Kato J. 2004. Chemotaxis proteins and transducers for aerotaxis in *Pseudomonas aeruginosa*. FEMS Microbiol Lett 231:247–252. doi:10.1016/S0378-1097(04)00009-6. 14987771

[48] Zhang L, Li S, Liu X, Wang Z, Jiang M, Wang R, Xie L, Liu Q, Xie X, Shang D, Li M, Wei Z, Wang Y, Fan C, Luo ZQ, Shen X. 2020. Sensing of autoinducer-2 by functionally distinct receptors in prokaryotes. Nat Commun 11:5371. doi:10.1038/s41467-020-19243-5. 33097715 PMC7584622

[49] Upadhyay AA, Fleetwood AD, Adebali O, Finn RD, Zhulin IB. 2016. Cache domains that are homologous to, but different from PAS domains comprise the largest superfamily of extracellular sensors in prokaryotes. PLoS Comput Biol 12:e1004862. doi:10.1371/journal.pcbi.1004862. 27049771 PMC4822843

[50] Webb BA, Karl Compton K, Castaneda Saldana R, Arapov TD, Keith Ray W, Helm RF, Scharf BE. 2017. *Sinorhizobium meliloti* chemotaxis to quaternary ammonium compounds is mediated by the chemoreceptor McpX. Mol Microbiol 103:333–346. doi:10.1111/mmi.13561. 27748981

[51] Reyes-Darias JA, Yang Y, Sourjik V, Krell T. 2015. Correlation between signal input and output in PctA and PctB amino acid chemoreceptor of *Pseudomonas aeruginosa*. Mol Microbiol 96:513–525. doi:10.1111/mmi.12953. 25641105

[52] Sourjik V, Berg HC. 2002. Receptor sensitivity in bacterial chemotaxis. Proc Natl Acad Sci USA 99:123–127. doi:10.1073/pnas.011589998. 11742065 PMC117525

[53] Sourjik V, Berg HC. 2002. Binding of the *Escherichia coli* response regulator CheY to its target measured *in vivo* by fluorescence resonance energy transfer. Proc Natl Acad Sci USA 99:12669–12674. doi:10.1073/pnas.192463199. 12232047 PMC130518

[54] Sourjik V, Vaknin A, Shimizu TS, Berg HC. 2007. *In vivo* measurement by FRET of pathway activity in bacterial chemotaxis. Methods Enzymol 423:365–391. doi:10.1016/S0076-6879(07)23017-4. 17609141

[55] Bi S, Pollard AM, Yang Y, Jin F, Sourjik V. 2016. Engineering hybrid chemotaxis receptors in bacteria. ACS Synth Biol 5:989–1001. doi:10.1021/acssynbio.6b00053. 27285081

[56] Shrestha M, Compton KK, Mancl JM, Webb BA, Brown AM, Scharf BE, Schubot FD. 2018. Structure of the sensory domain of McpX from *Sinorhizobium meliloti,* the first known bacterial chemotactic sensor for quaternary ammonium compounds. Biochem J 475:3949–3962. doi:10.1042/BCJ20180769. 30442721

[57] Machuca MA, Liu YC, Beckham SA, Gunzburg MJ, Roujeinikova A. 2016. The crystal structure of the tandem-PAS sensing domain of *Campylobacter jejuni* chemoreceptor Tlp1 suggests indirect mechanism of ligand recognition. J Struct Biol 194:205–213. doi:10.1016/j.jsb.2016.02.019. 26923153

[58] Ehrhardt MKG, Gerth ML, Johnston JM. 2021. Structure of a double CACHE chemoreceptor ligand-binding domain from *Pseudomonas syringae* provides insights into the basis of proline recognition. Biochem Biophys Res Commun 549:194–199. doi:10.1016/j.bbrc.2021.02.090. 33721671

[59] Cheung J, Hendrickson WA. 2008. Crystal structures of C4-dicarboxylate ligand complexes with sensor domains of histidine kinases DcuS and DctB. J Biol Chem 283:30256–30265. doi:10.1074/jbc.M805253200. 18701447 PMC2573060

[60] Wu R, Gu M, Wilton R, Babnigg G, Kim Y, Pokkuluri PR, Szurmant H, Joachimiak A, Schiffer M. 2013. Insight into the sporulation phosphorelay: crystal structure of the sensor domain of *Bacillus subtilis* histidine kinase, KinD. Protein Sci 22:564–576. doi:10.1002/pro.2237. 23436677 PMC3649258

[61] Velando F, Gavira JA, Rico-Jimenez M, Matilla MA, Krell T. 2020. Evidence for pentapeptide-dependent and independent CheB methylesterases. Int J Mol Sci 21:8459. doi:10.3390/ijms21228459. PMC7698151 33187094

[62] Brown DA. 2019. Acetylcholine and cholinergic receptors. Brain Neurosci Adv 3:2398212818820506. doi:10.1177/2398212818820506. 32166177 PMC7058246

[63] Wessler I, Kirkpatrick CJ, Racké K. 1999. The cholinergic ‘pitfall’: acetylcholine, a universal cell molecule in biological systems, including humans. Clin Exp Pharmacol Physiol 26:198–205. doi:10.1046/j.1440-1681.1999.03016.x. 10081614

[64] Kummer W, Lips KS, Pfeil U. 2008. The epithelial cholinergic system of the airways. Histochem Cell Biol 130:219–234. doi:10.1007/s00418-008-0455-2. 18566825 PMC2491704

[65] Mashimo M, Moriwaki Y, Misawa H, Kawashima K, Fujii T. 2021. Regulation of immune functions by non-neuronal acetylcholine (ACh) via muscarinic and nicotinic ACh receptors. Int J Mol Sci 22:6818. doi:10.3390/ijms22136818. 34202925 PMC8268711

[66] Grando SA, Kawashima K, Kirkpatrick CJ, Kummer W, Wessler I. 2015. Recent progress in revealing the biological and medical significance of the non-neuronal cholinergic system. Int Immunopharmacol 29:1–7. doi:10.1016/j.intimp.2015.08.023. 26362206

[67] Roshchina VV. 2016. New trends and perspectives in the evolution of neurotransmitters in microbial, plant, and animal cells. Adv Exp Med Biol 874:25–77. doi:10.1007/978-3-319-20215-0_2. 26589213

[68] Weinstein LI, Revuelta A, Pando RH. 2015. Catecholamines and acetylcholine are key regulators of the interaction between microbes and the immune system. Ann N Y Acad Sci 1351:39–51. doi:10.1111/nyas.12792. 26378438

[69] Shinozawa T, Fukunaga S. 1989. Acetylcholine inhibition of *Escherichia coli* chemotaxis for aspartate. Microbiol Immunol 33:689–692. doi:10.1111/j.1348-0421.1989.tb02019.x. 2674636

[70] Chet I, Henis Y, Mitchell R. 1973. Effect of biogenic amines and cannabinoids on bacterial chemotaxis. J Bacteriol 115:1215–1218. doi:10.1128/jb.115.3.1215-1218.1973. 4199511 PMC246374

[71] Bachas S, Eginton C, Gunio D, Wade H. 2011. Structural contributions to multidrug recognition in the multidrug resistance (MDR) gene regulator, BmrR. Proc Natl Acad Sci USA 108:11046–11051. doi:10.1073/pnas.1104850108. 21690368 PMC3131311

[72] Oswald C, Smits SH, Höing M, Sohn-Bösser L, Dupont L, Le Rudulier D, Schmitt L, Bremer E. 2008. Crystal structures of the choline/acetylcholine substrate-binding protein ChoX from *Sinorhizobium meliloti* in the liganded and unliganded-closed states. J Biol Chem 283:32848–32859. doi:10.1074/jbc.M806021200. 18779321

[73] Matilla MA, Ortega A, Krell T. 2021. The role of solute binding proteins in signal transduction. Comput Struct Biotechnol J 19:1786–1805. doi:10.1016/j.csbj.2021.03.029. 33897981 PMC8050422

[74] Dannelly KH, Liu Y, Ghosh SK. 2001. *Pseudomonas aeruginosa* corneal infection affects cholinergic enzymes in rat lacrimal gland. Arch Microbiol 177:47–53. doi:10.1007/s00203-001-0360-8. 11797044

[75] Pesin SR, Candia OA. 1982. Acetylcholine concentration and its role in ionic transport by the corneal epithelium. Invest Ophthalmol Vis Sci 22:651–659. 6978868

[76] Matilla MA, Krell T. 2018. The effect of bacterial chemotaxis on host infection and pathogenicity. FEMS Microbiol Rev doi:10.1093/femsre/fux052. 29069367

[77] Rossi E, Falcone M, Molin S, Johansen HK. 2018. High-resolution *in situ* transcriptomics of *Pseudomonas aeruginosa* unveils genotype independent patho-phenotypes in cystic fibrosis lungs. Nat Commun 9:3459. doi:10.1038/s41467-018-05944-5. 30150613 PMC6110831

[78] Cornforth DM, Dees JL, Ibberson CB, Huse HK, Mathiesen IH, Kirketerp-Møller K, Wolcott RD, Rumbaugh KP, Bjarnsholt T, Whiteley M. 2018. *Pseudomonas aeruginosa* transcriptome during human infection. Proc Natl Acad Sci USA 115:E5125–E5134. doi:10.1073/pnas.1717525115. 29760087 PMC5984494

[79] Damron FH, Oglesby-Sherrouse AG, Wilks A, Barbier M. 2016. Dual-seq transcriptomics reveals the battle for iron during *Pseudomonas aeruginosa* acute murine pneumonia. Sci Rep 6:39172. doi:10.1038/srep39172. 27982111 PMC5159919

[80] Jumper J, Evans R, Pritzel A, Green T, Figurnov M, Ronneberger O, Tunyasuvunakool K, Bates R, Žídek A, Potapenko A, Bridgland A, Meyer C, Kohl SAA, Ballard AJ, Cowie A, Romera-Paredes B, Nikolov S, Jain R, Adler J, Back T, Petersen S, Reiman D, Clancy E, Zielinski M, Steinegger M, Pacholska M, Berghammer T, Bodenstein S, Silver D, Vinyals O, Senior AW, Kavukcuoglu K, Kohli P, Hassabis D. 2021. Highly accurate protein structure prediction with AlphaFold. Nature 596:583–589. doi:10.1038/s41586-021-03819-2. 34265844 PMC8371605

[81] Orr AA, Yang J, Sule N, Chawla R, Hull KG, Zhu M, Romo D, Lele PP, Jayaraman A, Manson MD, Tamamis P. 2020. Molecular mechanism for attractant signaling to DHMA by *E. coli* Tsr. Biophys J 118:492–504. doi:10.1016/j.bpj.2019.11.3382. 31839263 PMC6976796

[82] Sule N, Pasupuleti S, Kohli N, Menon R, Dangott LJ, Manson MD, Jayaraman A. 2017. The norepinephrine metabolite 3,4-dihydroxymandelic acid is produced by the commensal microbiota and promotes chemotaxis and virulence gene expression in enterohemorrhagic *Escherichia coli*. Infect Immun 85:e00431-17. doi:10.1128/IAI.00431-17. 28717028 PMC5607413

[83] Pasupuleti S, Sule N, Manson MD, Jayaraman A. 2018. Conversion of norepinephrine to 3,4-dihdroxymandelic acid in *Escherichia coli* requires the QseBC quorum-sensing system and the FeaR transcription factor. J Bacteriol 200:e00564-17. doi:10.1128/JB.00564-17. 29038253 PMC5717157

[84] Clarke MB, Hughes DT, Zhu C, Boedeker EC, Sperandio V. 2006. The QseC sensor kinase: a bacterial adrenergic receptor. Proc Natl Acad Sci USA 103:10420–10425. doi:10.1073/pnas.0604343103. 16803956 PMC1482837

[85] Roychoudhury A. 2020. Neurotransmitter acetylcholine comes to the plant rescue. J Mol Cell Biol Forecast 3:1019.

[86] Tretyn A, Łukasiewicz-Rutkowska H, Kopcewicz J. 1997. Isolation, purification and identification of acetylcholine in *Pharbitis nil* seedlings. Acta Physiol Plant 19:303–309. doi:10.1007/s11738-997-0006-9.

[87] Hartmann E, Kilbinger H. 1974. Occurrence of light-dependent acetylcholine concentrations in higher plants. Experientia 30:1387–1388. doi:10.1007/BF01919649. 4442525

[88] Bond SR, Naus CC. 2012. RF-Cloning.org: an online tool for the design of restriction-free cloning projects. Nucleic Acids Res 40:W209–W213. doi:10.1093/nar/gks396. 22570410 PMC3394257

[89] Abril MA, Michan C, Timmis KN, Ramos JL. 1989. Regulator and enzyme specificities of the TOL plasmid-encoded upper pathway for degradation of aromatic hydrocarbons and expansion of the substrate range of the pathway. J Bacteriol 171:6782–6790. doi:10.1128/jb.171.12.6782-6790.1989. 2687253 PMC210577

[90] González-Ramírez LA, Ruiz-Martínez CR, Estremera-Andújar RA, Nieves-Marrero CA, García-Caballero A, Gavira JA, López-Garriga J, García-Ruiz JM. 2017. Efficient screening methodology for protein crystallization based on the counter-diffusion technique. Cryst Growth Des 17:6780–6786. doi:10.1021/acs.cgd.7b01353.

[91] Kabsch W. 2010. XDS. Acta Crystallogr D Biol Crystallogr 66:125–132. doi:10.1107/S0907444909047337. 20124692 PMC2815665

[92] Evans PR, Murshudov GN. 2013. How good are my data and what is the resolution? Acta Crystallogr D Biol Crystallogr 69:1204–1214. doi:10.1107/S0907444913000061. 23793146 PMC3689523

[93] Collaborative Computational Project, No. 4. 1994. The CCP4 suite: programs for protein crystallography. Acta Crystallogr D Biol Crystallogr 50:760–763. doi:10.1107/S0907444994003112. 15299374

[94] Vagin A, Teplyakov A. 2010. Molecular replacement with MOLREP. Acta Crystallogr D Biol Crystallogr 66:22–25. doi:10.1107/S0907444909042589. 20057045

[95] Kallberg M, Wang H, Wang S, Peng J, Wang Z, Lu H, Xu J. 2012. Template-based protein structure modeling using the RaptorX web server. Nat Protoc 7:1511–1522. doi:10.1038/nprot.2012.085. 22814390 PMC4730388

[96] Baek M, DiMaio F, Anishchenko I, Dauparas J, Ovchinnikov S, Lee GR, Wang J, Cong Q, Kinch LN, Schaeffer RD, Millan C, Park H, Adams C, Glassman CR, DeGiovanni A, Pereira JH, Rodrigues AV, van Dijk AA, Ebrecht AC, Opperman DJ, Sagmeister T, Buhlheller C, Pavkov-Keller T, Rathinaswamy MK, Dalwadi U, Yip CK, Burke JE, Garcia KC, Grishin NV, Adams PD, Read RJ, Baker D. 2021. Accurate prediction of protein structures and interactions using a three-track neural network. Science 373:871–876. doi:10.1126/science.abj8754. 34282049 PMC7612213

[97] Robin X, Haas J, Gumienny R, Smolinski A, Tauriello G, Schwede T. 2021. Continuous Automated Model EvaluatiOn (CAMEO)-Perspectives on the future of fully automated evaluation of structure prediction methods. Proteins 89:1977–1986. doi:10.1002/prot.26213. 34387007 PMC8673552

[98] Afonine PV, Mustyakimov M, Grosse-Kunstleve RW, Moriarty NW, Langan P, Adams PD. 2010. Joint X-ray and neutron refinement with phenix.refine. Acta Crystallogr D Biol Crystallogr 66:1153–1163. doi:10.1107/S0907444910026582. 21041930 PMC2967420

[99] Murshudov GN, Skubak P, Lebedev AA, Pannu NS, Steiner RA, Nicholls RA, Winn MD, Long F, Vagin AA. 2011. REFMAC5 for the refinement of macromolecular crystal structures. Acta Crystallogr D Biol Crystallogr 67:355–367. doi:10.1107/S0907444911001314. 21460454 PMC3069751

[100] Emsley P, Lohkamp B, Scott WG, Cowtan K. 2010. Features and development of Coot. Acta Crystallogr D Biol Crystallogr 66:486–501. doi:10.1107/S0907444910007493. 20383002 PMC2852313

[101] Painter J, Merritt EA. 2006. TLSMD web server for the generation of multi-group TLS models. J Appl Crystallogr 39:109–111. doi:10.1107/S0021889805038987.

[102] Chen VB, Arendall WB III, Headd JJ, Keedy DA, Immormino RM, Kapral GJ, Murray LW, Richardson JS, Richardson DC. 2010. MolProbity: all-atom structure validation for macromolecular crystallography. Acta Crystallogr D Biol Crystallogr 66:12–21. doi:10.1107/S0907444909042073. 20057044 PMC2803126

[103] Velankar S, Alhroub Y, Alili A, Best C, Boutselakis HC, Caboche S, Conroy MJ, Dana JM, van Ginkel G, Golovin A, Gore SP, Gutmanas A, Haslam P, Hirshberg M, John M, Lagerstedt I, Mir S, Newman LE, Oldfield TJ, Penkett CJ, Pineda-Castillo J, Rinaldi L, Sahni G, Sawka G, Sen S, Slowley R, Sousa da Silva AW, Suarez-Uruena A, Swaminathan GJ, Symmons MF, Vranken WF, Wainwright M, Kleywegt GJ. 2011. PDBe: Protein Data Bank in Europe. Nucleic Acids Res 39:D402–D410. doi:10.1093/nar/gkq985. 21045060 PMC3013808

[104] Bi S, Jin F, Sourjik V. 2018. Inverted signaling by bacterial chemotaxis receptors. Nat Commun 9:2927. doi:10.1038/s41467-018-05335-w. 30050034 PMC6062612

[105] Si G, Yang W, Bi S, Luo C, Ouyang Q. 2012. A parallel diffusion-based microfluidic device for bacterial chemotaxis analysis. Lab Chip 12:1389–1394. doi:10.1039/c2lc21219f. 22361931

[106] Laskowski RA, Swindells MB. 2011. LigPlot+: multiple ligand-protein interaction diagrams for drug discovery. J Chem Inf Model 51:2778–2786. doi:10.1021/ci200227u. 21919503

[107] Combet C, Blanchet C, Geourjon C, Deléage G. 2000. NPS@: network protein sequence analysis. Trends Biochem Sci 25:147–150. doi:10.1016/s0968-0004(99)01540-6. 10694887

[108] Holm L. 2020. Using Dali for protein structure comparison. Methods Mol Biol 2112:29–42. doi:10.1007/978-1-0716-0270-6_3. 32006276

