# Supplementary figures and images for: Chemotaxis of the Human Pathogen Pseudomonas aeruginosa to the Neurotransmitter Acetylcholine
Source: mBio. 2022 Mar 7;13(2):e03458-21. doi: 10.1128/mbio.03458-21 (PMC9040839; doi:10.1128/mbio.03458-21)

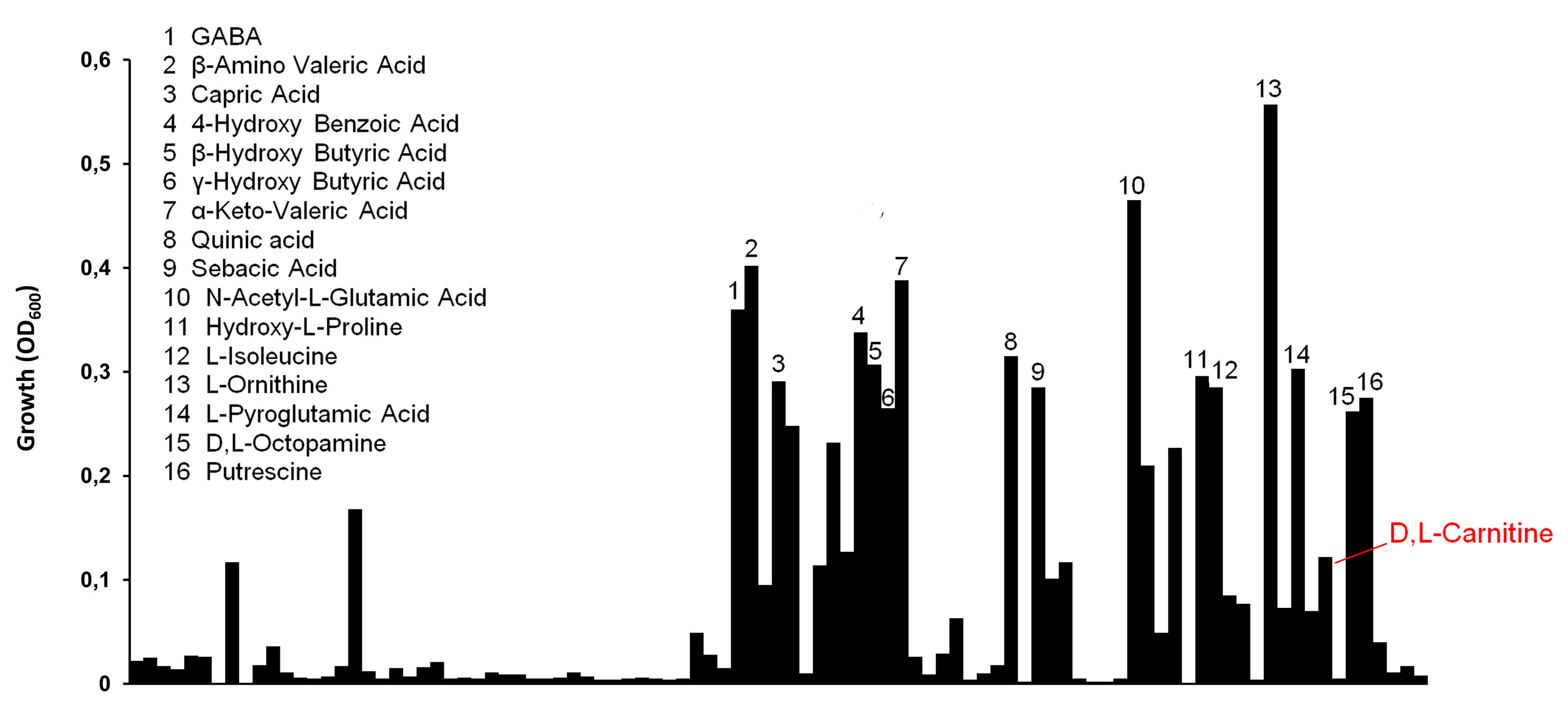

Supplement: FIG S1 [file mbio.03458-21-sf001.jpg]

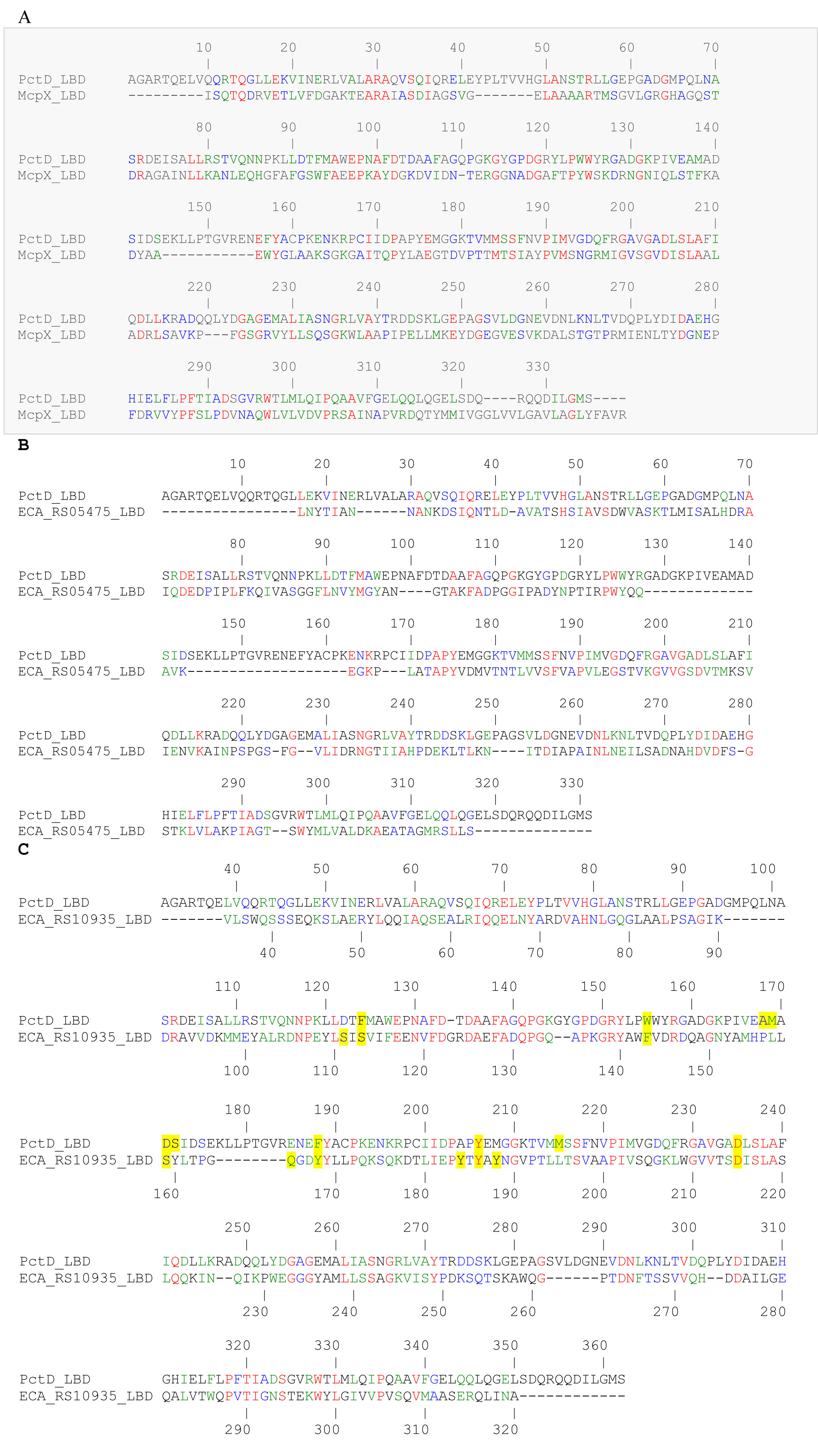

Supplement: FIG S2 [file mbio.03458-21-sf002.jpg]

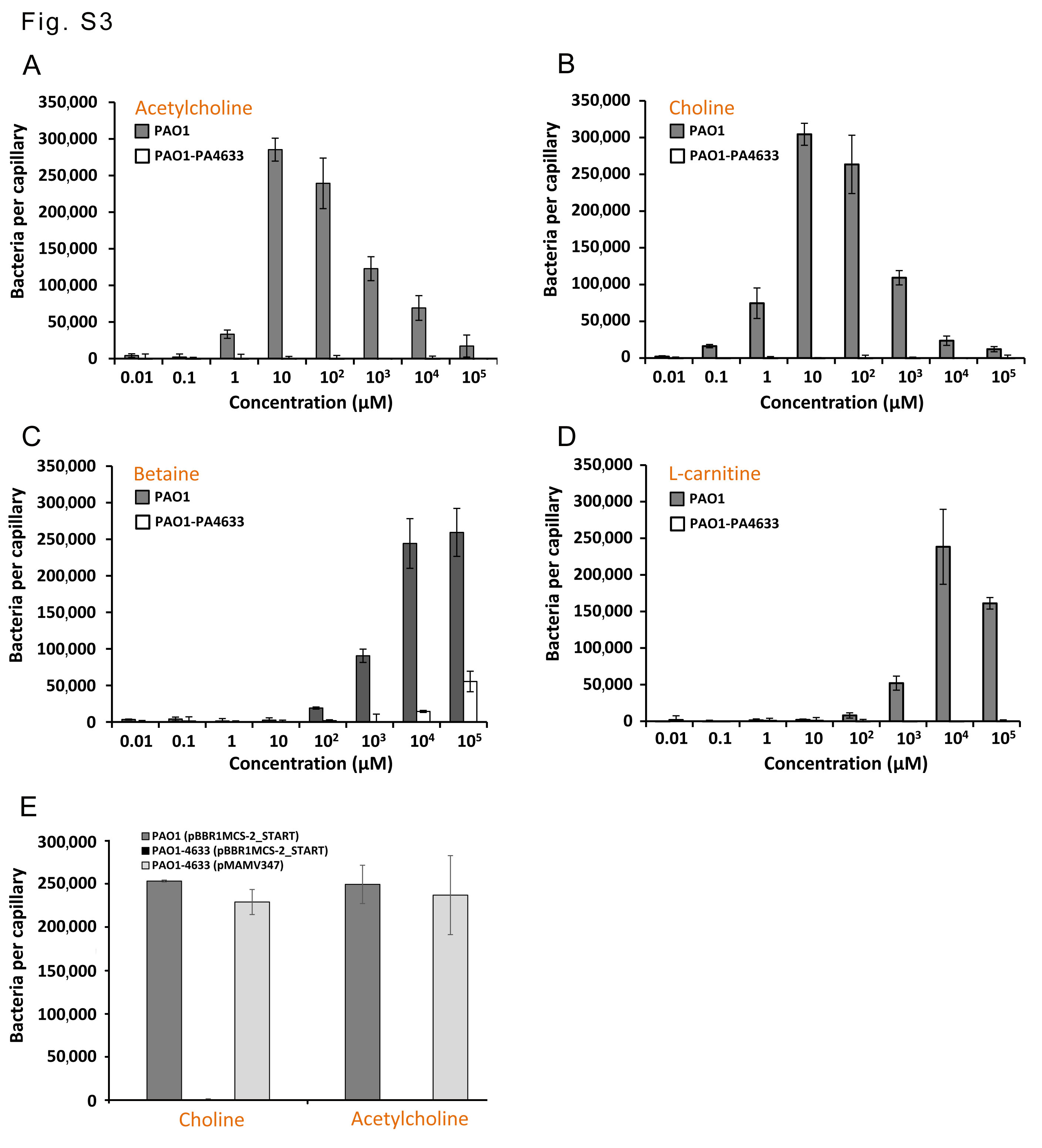

Supplement: FIG S3 [file mbio.03458-21-sf003.jpg]

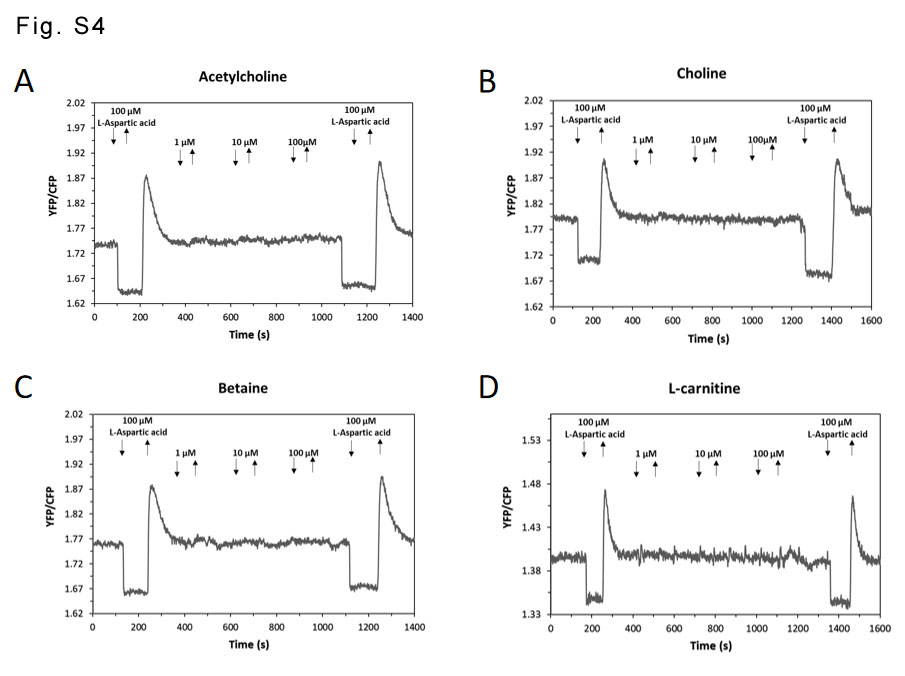

Supplement: FIG S4 [file mbio.03458-21-sf004.jpg]

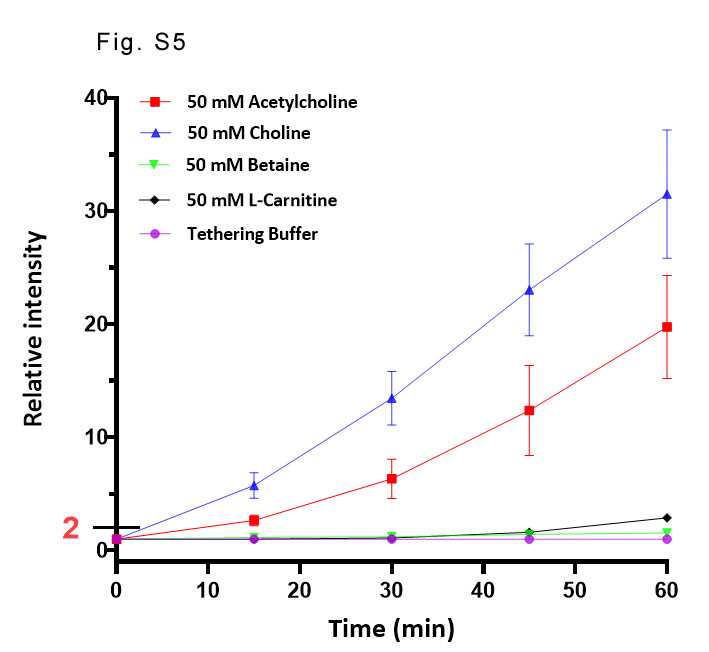

Supplement: FIG S5 [file mbio.03458-21-sf005.jpg]

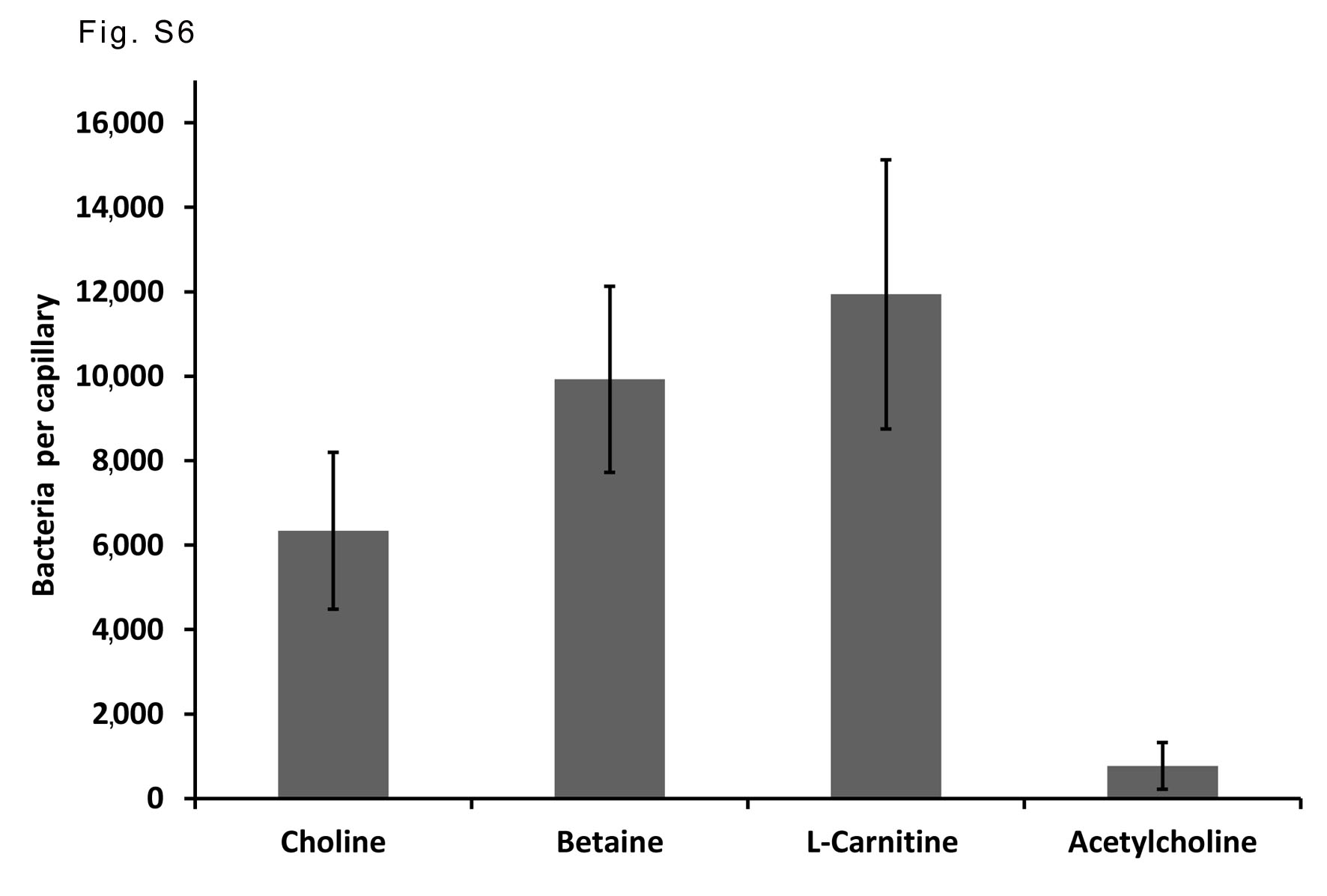

Supplement: FIG S6 [file mbio.03458-21-sf006.jpg]

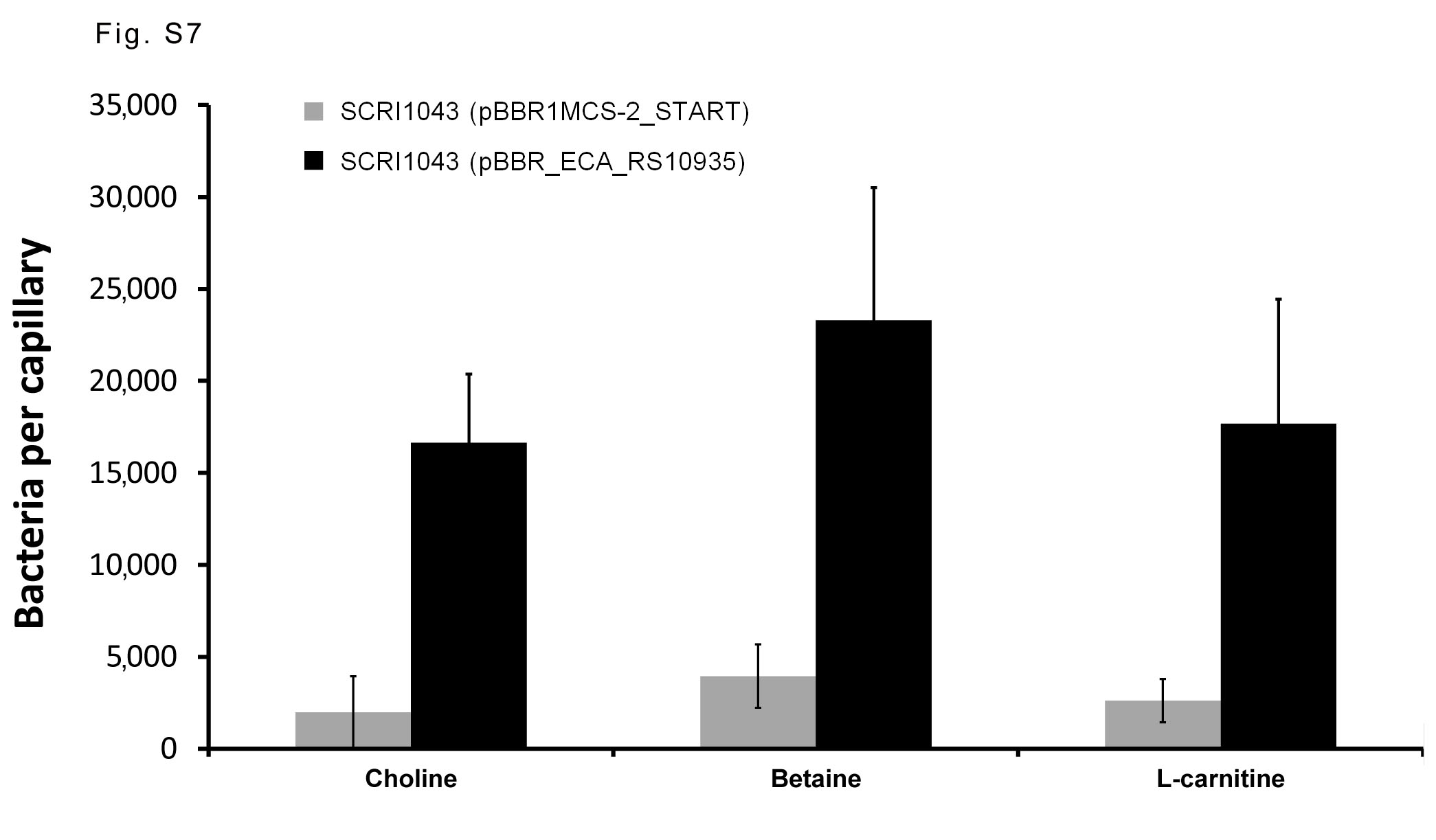

Supplement: FIG S7 [file mbio.03458-21-sf007.jpg]
