# Supplementary material for: Chemotaxis of the Human Pathogen Pseudomonas aeruginosa to the Neurotransmitter Acetylcholine
Source: mBio. 2022 Mar 7;13(2):e03458-21. doi: 10.1128/mbio.03458-21 (PMC9040839; doi:10.1128/mbio.03458-21)
Supplement: TABLE S1 [file mbio.03458-21-st001.docx]

**Table S1) Structural alignment of the PctD-LBD structure (in complex with acetylcholine) and the PacA-LBD structure (in complex with betaine) with structures deposited in the Protein Data Bank**. Shown are the top 15 structures according to the Z-score. Alignments were made with the DALI server (Holm (2020) Methods Mol Biol*.* 2112, 29).

| PDB ID | Name | Sensor protein family | Ligands | Bacterial species | Z-score | rmsd | nres | % id |
| --- | --- | --- | --- | --- | --- | --- | --- | --- |
| **PctD-LBD** | | | | | | | | |
| 6fu4 | TlpQ | CR | Histamine, polyamines | *P. aeruginosa* | 28.1 | 2.8 | 297 | 25 |
| 6pzj |  | CR | Unknown | *Leptospira interrogans* | 24.8 | 2.4 | 264 | 26 |
| 3lib | mmHK1S-Z3 | SK | Unknown | *Methanosarcina mazei* | 24.3 | 3.0 | 271 | 23 |
| 6d8v | McpX | CR | Quaternary amines | *Sinorhizobium meliloti* | 23.5 | 3.6 | 270 | 19 |
| 4wy9 | Tlp1 | CR | Asp | *Campylobacter jejuni* | 21.3 | 4.3 | 285 | 13 |
| 6e0a | TlpA | CR | Unknown | *Helicobacter pylori* | 19.5 | 3.9 | 262 | 16 |
| 6mni | PscC | CR | Pro | *P. syringae* | 18.7 | 3.3 | 259 | 13 |
| 5ere |  | Extracellular  receptor |  | Desulfohalobium retbaense | 18.0 | 3.9 | 540 | 12 |
| 5lt9 | PctB | CR | Gln and other amino acids | *P. aeruginosa* | 17.1 | 3.7 | 254 | 15 |
| 4xmq | Tlp3 | CR | Amino acids and other compounds | *C. jejuni* | 16.6 | 3.4 | 254 | 9 |
| 3lic | soHK1S-Z6 | SK | Unknown | *Shewanella oneidensis* | 15.7 | 4.4 | 265 | 18 |
| 3lid | vpHK1S-Z8 | SK | Unknown | *Vibrio paraheamolyticus* | 15.3 | 4.9 | 279 | 11 |
| 3lif | rpHK1S-Z16 | SK | Unknown | *Rhodopseudomonas palustris* | 15.2 | 3.5 | 243 | 12 |
| 3by9 | DctB | SK | Succinate | *Vibrio cholerae* | 14.6 | 3.8 | 259 | 9 |
| 4jgo | KinD | SK | Pyruvate, propionate, butyrate, autoinducer-2 | *Bacillus subtilis* | 14.4 | 3.7 | 210 | 9 |
| **PacA-LBD** | | | | | | | | |
| 6pzj |  | CR | Unknown | *Leptospira interrogans* | 28.0 | 2.3 | 264 | 22 |
| 3lib | mmHK1S-Z3 | SK | Unknown | *Methanosarcina mazei* | 27.5 | 2.8 | 271 | 26 |
| 6fu4 | TlpQ | CR | Histamine, polyamines | *P. aeruginosa* | 27.0 | 2.3 | 297 | 26 |
| 6d8v | McpX | CR | Quaternary amines | *Sinorhizobium meliloti* | 24.8 | 3.2 | 270 | 22 |
| 4wy9 | Tlp1 | CR | Asp | *Campylobacter jejuni* | 21.5 | 4.4 | 285 | 13 |
| 3lic | soHK1S-Z6 | SK | Unknown | *Shewanella oneidensis* | 20.2 | 4.4 | 265 | 18 |
| 6e0a | TlpA | CR | Unknown | *Helicobacter pylori* | 19.9 | 3.3 | 262 | 14 |
| 6mni | PscC | CR | Pro | *P. syringae* | 19.7 | 3.1 | 259 | 19 |
| 5lt9 | PctB | CR | Gln and other amino acids | *P. aeruginosa* | 19.4 | 3.5 | 254 | 25 |
| 5ere |  | Extracellular  receptor |  | *Desulfohalobium retbaense* | 18.6 | 3.2 | 540 | 8 |
| 4xmq | Tlp3 | CR | Amino acids and other compounds | *C. jejuni* | 16.6 | 3.4 | 254 | 12 |
| 3lif | rpHK1S-Z16 | SK | Unknown | *Rhodopseudomonas palustris* | 16.3 | 3.3 | 243 | 13 |
| 7k5n |  | Diguanylate cyclase | Pro | *Aeromonas caviae* | 16.3 | 3.7 | 238 | 20 |
| 2zbb | DctB | SK | Succinate | *Escherichia coli* | 15.0 | 3.8 | 255 | 12 |
| 4jgo | KinD | SK | Pyruvate, propionate, butyrate, autoinducer-2 | *Bacillus subtilis* | 14.4 | 3.6 | 210 | 11 |

CR: chemoreceptor; SK: sensor kinase
