# Supplementary material for: Chemotaxis of the Human Pathogen Pseudomonas aeruginosa to the Neurotransmitter Acetylcholine
Source: mBio. 2022 Mar 7;13(2):e03458-21. doi: 10.1128/mbio.03458-21 (PMC9040839; doi:10.1128/mbio.03458-21)
Supplement: TABLE S2 [file mbio.03458-21-st002.docx]

**Table S2) Strains, plasmids and oligonucleotides used in this study.**

| **Strains and plasmids** | | **Genotype or relevant characteristics^a^** | | **Ref.** |
| --- | --- | --- | --- | --- |
| **Strains** | |  | |  |
| *Escherichia coli* BL21(DE3) | | F^–^ *ompT* *gal* *dcm* *lon* *hsdS_B_*(*r_B_*^–^*m_B_*^–^) λ(DE3 [*lacI* *lacUV5*-*T7p07* *ind1* *sam7* *nin5*]) [*malB*^+^]_K-12_(λ^S^) | | (1) |
| *E. coli* BL21-AI | | F- *ompT hsdS*_B_ (r_B_^-^m_B_^-^) *gal dcm araB*::*T7RNAP-tetA* | | Invitrogen |
| *E. coli* DH5α | | F^–^ *endA1* *glnV44* *thi-1*  *recA1*  *relA1*  *gyrA96 deoR* *nupG* *purB20* φ80d*lacZ*ΔM15 Δ(*lacZYA-argF*)U169, hsdR17(*r_K_*^–^*m_K_*^+^), λ^–^ | | (2) |
| *E. coli* CC118λ*pir* | | *araD* Δ(*ara*, *leu*) Δ*lacZ74 phoA20 galK thi-1 rspE rpoB argE recA1* λ*pir* | | (3) |
| *E. coli* HH26 | | Mobilizing strain for conjugal transfer | | (4) |
| *E. coli* K12 | | F^-^ lambda^-^ *ilvG* *rfb*-50 *rph*-1 | | (5) |
| *E. coli* UU1250 | | Derivative of RP437, Δ*aer*Δ*tsr*Δ*(tar-tap)*Δ*trg* | | (6) |
| *E. coli* VS181 | | Derivative of RP437; Δ(*cheYcheZ*)Δ*aer*Δ*tsr*Δ(*tar-tap*) Δ*trg* | | (7) |
| *Pseudomonas aeruginosa* PAO1 | | Wild type | | (8) |
| *P. aeruginosa* PAO1-PA4633 | | PAO1 transposon mutant *pa4633*::IS*lacZ*/hah; Tc^R^ | | (9) |
| *Pectobacterium atrosepticum* SCRI1043 | | Wild type, plant pathogen | | (4) |
| *P. atrosepticum* SCRI1043-ECA_RS10935 | | Deletion mutant of *ECA_RS10935* | | This study |
| *Pantoea agglomerans* 9Rz4 | | Wild type, oilseed rape rhizosphere isolate | | (10) |
| *Serratia plymuthic*a A153 | | Wild type, wheat rhizosphere isolate | | (11) |
| *Pseudomonas stutzeri* AN10 PSC306 | | Wild type, polluted marine sediment isolate | | (12) |
| *Agrobacterium tumefaciens* C58 | | Wild-type, plant pathogen | | (13) |
| *Dickeya solani* MK10 | | Wild-type, plant pathogen | | (14) |
| *P. savastanoi pv. savastanoi* NCPPB 3335 | | Wild-type, plant pathogen | | (15) |
| *Pseudomonas putida* KT2440 | | Wild-type, non-pathogenic soil bacterium | | (16) |
| *Salmonella enterica* sv. Typhimurium ATCC14028 | | Wild-type, human and animal pathogen | | (17) |
| **Plasmids** | | | | |
| pET28b(+) | | Km^R^, protein expression plasmid | | Novagen |
| pET28_PA4633-LBD | | Km^R^; pET28b(+) derivative containing a DNA fragment encoding PA4633-LBD | | This study |
| pET28_ECA_RS10935-LBD | | Km^R^; pET28b(+) derivative containing a DNA fragment encoding ECA_RS10935-LBD | | This study |
| pBBR1MCS-2_START | | Km^R^; *oriRK2 mobRK2* | | (18) |
| pBBR_ECA_RS10935 | | Km^R^; a 2.0-kb PCR fragment containing the RS10935 gene cloned into the NdeI/EcoRI sites of pBBR1MCS-2_START | | This study |
| pMAMV347 | | Km^R^; a 2.2-kb PCR fragment containing the *pa4633* gene cloned into the NdeI/EcoRI sites of pBBR1MCS-2_START | | This study |
| pKG116 | | Cm^R^; Protein expression plasmid | | (19) |
| pVS1743 | | Cm^R^; pKG116 derivative containing a DNA fragment encoding PctD [1-435]-Tar [267-553]; | | This study |
| pSB13 | | Cm^R^; pKG116 derivative, Tar expression plasmid | | (20) |
| pVS88 | | Ap^R^; CheY-EYFP / CheZ-ECFP expression plasmid | | (7) |
| pOB30 | | Ap^R^; GFP expression plasmid | | (21) |
| **Oligonucleotides** | | | | |
| **Name** | **Sequence (5’-3’)** | | **Purpose** | |
| PA4633-LBD-f | GGAATTCCATATGGCCGGCGCGCGAACGCA | | Construction of pET28_PA4633-LBD | |
| PA4633-LBD-r | CCGCTCGAGTCACATGCCGAGGATATCCTGCT | |  |  |
| ECA_RS10935-LBD-f | CCGCGCGGCAGCCATATGAGCTGGCAGTCTAGTAGTGA | | Construction of pET28_ECA_RS10935-LBD | |
| ECA_RS10935-LBD-r | TCGACGGAGCTCGAATTCGGATCTCACAACTGGCGCTCAGACG | |  |  |
| PA4633-NdeI-f | TAATCATATGAAGCTCAAGTCGATCCAGTT | | Construction of pMAMV347 | |
| PA4633-EcoRI-r | TAATGAATTCCTGATAAGCCTGCTCGCCAAG | |  |  |
| 1F-ECA_RS10935 | taatgccatatggccaaacaattaatac | | Construction of pBBR_ECA_RS10935 | |
| 1R-ECA_RS10935 | taatggatccccttatggtcgccgggttac | |  |  |
| PA4633-NdeI-f | AAACATATGATGAAGCTCAAGTCGATCCAG | | Construction of pVS1743 | |
| PA4633-r | CCTTCGCGGACATGGCCGATCATGTTCTGCAGTTTG | |  |  |
| Tar-PA4633-f | GATCGGCCATGTCCGCGAAGGTTCAGAT | |  |  |
| Tar-BamHI-r | GGATCCTCAAAATGTTTCCCAGTTT | |  |  |

^a^Ap, ampicillin; Km, kanamycin; Tc, tetracycline; Sm, streptomycin; Cm, chloramphenicol.

**References**

1. Jeong H, Barbe V, Lee CH, Vallenet D, Yu DS, Choi SH, Couloux A, Lee SW, Yoon SH, Cattolico L, Hur CG, Park HS, Segurens B, Kim SC, Oh TK, Lenski RE, Studier FW, Daegelen P, Kim JF. 2009. Genome sequences of *Escherichia coli* B strains REL606 and BL21(DE3). J Mol Biol 394:644-52.

2. Woodcock DM, Crowther PJ, Doherty J, Jefferson S, DeCruz E, Noyer-Weidner M, Smith SS, Michael MZ, Graham MW. 1989. Quantitative evaluation of *Escherichia coli* host strains for tolerance to cytosine methylation in plasmid and phage recombinants. Nucleic Acids Res 17:3469-78.

3. Herrero M, de Lorenzo V, Timmis KN. 1990. Transposon vectors containing non-antibiotic resistance selection markers for cloning and stable chromosomal insertion of foreign genes in gram-negative bacteria. J Bacteriol 172:6557-67.

4. Kaniga K, Delor I, Cornelis GR. 1991. A wide-host-range suicide vector for improving reverse genetics in gram-negative bacteria: inactivation of the blaA gene of Yersinia enterocolitica. Gene 109:137-41.

5. Blattner FR, Plunkett G, 3rd, Bloch CA, Perna NT, Burland V, Riley M, Collado-Vides J, Glasner JD, Rode CK, Mayhew GF, Gregor J, Davis NW, Kirkpatrick HA, Goeden MA, Rose DJ, Mau B, Shao Y. 1997. The complete genome sequence of Escherichia coli K-12. Science 277:1453-62.

6. Ames P, Studdert CA, Reiser RH, Parkinson JS. 2002. Collaborative signaling by mixed chemoreceptor teams in Escherichia coli. Proc Natl Acad Sci U S A 99:7060-5.

7. Sourjik V, Berg HC. 2004. Functional interactions between receptors in bacterial chemotaxis. Nature 428:437-41.

8. Stover CK, Pham XQ, Erwin AL, Mizoguchi SD, Warrener P, Hickey MJ, Brinkman FS, Hufnagle WO, Kowalik DJ, Lagrou M, Garber RL, Goltry L, Tolentino E, Westbrock-Wadman S, Yuan Y, Brody LL, Coulter SN, Folger KR, Kas A, Larbig K, Lim R, Smith K, Spencer D, Wong GK, Wu Z, Paulsen IT, Reizer J, Saier MH, Hancock RE, Lory S, Olson MV. 2000. Complete genome sequence of Pseudomonas aeruginosa PAO1, an opportunistic pathogen. Nature 406:959-64.

9. Jacobs MA, Alwood A, Thaipisuttikul I, Spencer D, Haugen E, Ernst S, Will O, Kaul R, Raymond C, Levy R, Chun-Rong L, Guenthner D, Bovee D, Olson MV, Manoil C. 2003. Comprehensive transposon mutant library of Pseudomonas aeruginosa. Proc Natl Acad Sci U S A 100:14339-44.

10. Berg G, Roskot N, Steidle A, Eberl L, Zock A, Smalla K. 2002. Plant-dependent genotypic and phenotypic diversity of antagonistic rhizobacteria isolated from different Verticillium host plants. Appl Environ Microbiol 68:3328-38.

11. Matilla MA, Drew A, Udaondo Z, Krell T, Salmond GP. 2016. Genome Sequence of *Serratia plymuthica* A153, a Model Rhizobacterium for the Investigation of the Synthesis and Regulation of Haterumalides, Zeamine, and Andrimid. Genome Announc 4.

12. Brunet-Galmes I, Busquets A, Pena A, Gomila M, Nogales B, Garcia-Valdes E, Lalucat J, Bennasar A, Bosch R. 2012. Complete genome sequence of the naphthalene-degrading bacterium Pseudomonas stutzeri AN10 (CCUG 29243). J Bacteriol 194:6642-3.

13. Wood DW, Setubal JC, Kaul R, Monks DE, Kitajima JP, Okura VK, Zhou Y, Chen L, Wood GE, Almeida NF, Jr., Woo L, Chen Y, Paulsen IT, Eisen JA, Karp PD, Bovee D, Sr., Chapman P, Clendenning J, Deatherage G, Gillet W, Grant C, Kutyavin T, Levy R, Li MJ, McClelland E, Palmieri A, Raymond C, Rouse G, Saenphimmachak C, Wu Z, Romero P, Gordon D, Zhang S, Yoo H, Tao Y, Biddle P, Jung M, Krespan W, Perry M, Gordon-Kamm B, Liao L, Kim S, Hendrick C, Zhao ZY, Dolan M, Chumley F, Tingey SV, Tomb JF, Gordon MP, Olson MV, et al. 2001. The genome of the natural genetic engineer Agrobacterium tumefaciens C58. Science 294:2317-23.

14. Pritchard L, Humphris S, Baeyen S, Maes M, Van Vaerenbergh J, Elphinstone J, Saddler G, Toth I. 2013. Draft Genome Sequences of Four Dickeya dianthicola and Four Dickeya solani Strains. Genome Announc 1.

15. Rodriguez-Palenzuela P, Matas IM, Murillo J, Lopez-Solanilla E, Bardaji L, Perez-Martinez I, Rodriguez-Moskera ME, Penyalver R, Lopez MM, Quesada JM, Biehl BS, Perna NT, Glasner JD, Cabot EL, Neeno-Eckwall E, Ramos C. 2010. Annotation and overview of the Pseudomonas savastanoi pv. savastanoi NCPPB 3335 draft genome reveals the virulence gene complement of a tumour-inducing pathogen of woody hosts. Environ Microbiol 12:1604-20.

16. Espinosa-Urgel M, Ramos JL. 2004. Cell density-dependent gene contributes to efficient seed colonization by Pseudomonas putida KT2440. Appl Environ Microbiol 70:5190-8.

17. Romling U, Bian Z, Hammar M, Sierralta WD, Normark S. 1998. Curli fibers are highly conserved between Salmonella typhimurium and Escherichia coli with respect to operon structure and regulation. J Bacteriol 180:722-31.

18. Obranic S, Babic F, Maravic-Vlahovicek G. 2013. Improvement of pBBR1MCS plasmids, a very useful series of broad-host-range cloning vectors. Plasmid 70:263-7.

19. Buron-Barral MC, Gosink KK, Parkinson JS. 2006. Loss- and gain-of-function mutations in the F1-HAMP region of the Escherichia coli aerotaxis transducer Aer. J Bacteriol 188:3477-86.

20. Bi S, Jin F, Sourjik V. 2018. Inverted signaling by bacterial chemotaxis receptors. Nat Commun 9:2927.

21. Bi S, Pollard AM, Yang Y, Jin F, Sourjik V. 2016. Engineering Hybrid Chemotaxis Receptors in Bacteria. ACS Synth Biol 5:989-1001.
