# Supplementary material for: Chemotaxis of the Human Pathogen Pseudomonas aeruginosa to the Neurotransmitter Acetylcholine
Source: mBio. 2022 Mar 7;13(2):e03458-21. doi: 10.1128/mbio.03458-21 (PMC9040839; doi:10.1128/mbio.03458-21)
Supplement: TABLE S3 [file mbio.03458-21-st003.docx]

## Table S3) Crystallization conditions, data collection and refinement statistics of the PctD-LBD and PacA-LBD three-dimensional structures.

|  | | PctD-LBD/choline | PctD-LBD/acetylcholine | PacA-LBD/betaine |
| --- | --- | --- | --- | --- |
| Crystallization conditions | | | | |
|  | 30% (w/v) PEG 4K, 0.2 M NH_4_ acetate, 0.1 M Na acetate, pH 4.6 | | 30% (w/v) PEG 4K, 0.2 M NH_4_ acetate, 0.1 M Na acetate, pH 4.6 | 30% (w/v) PEG 4K, 0.2 M Na acetate, 0.1 M Tris hydrochloride, pH 8.5 |
| Data collection | | | | |
| PDB ID. | 7PRQ | | 7PRR | 7PSG |
| Beam Line | ID23-1 | | Xaloc | ID30A-3 |
| Space group | P 21 21 21 | | P 21 21 21 | P 1 21 1 |
| Unit cell a, b, c (Å) | 56.14, 104.86, 118.60 | | 62.29, 102.65, 104.85 | 80.78, 83.44, 94.91 |
| ASU | 2 | | 2 | 4 |
| Resolution (Å) ^*^ | 59.3-2.0 (2.07-2.0) | | 47.48-1.8 (1.86-1.8) | 39.99-1.91 (1.98-1.91) |
| Unique reflections^*^ | 47,974 (4,694) | | 62,195 (6,172) | 91,902 (9,315) |
| Multiplicity^*^ | 4.5 (4.5) | | 4.5 (4.6) | 2.9 (3.1) |
| Completeness (%)^*^ | 99.69 (99.62) | | 98.62 (99.42) | 98.01 (98.75) |
| I/σ_I_ ^*^ | 11.25 (1.81) | | 12.00 (1.32) | 10.95 (0.95) |
| Wilson B-factor | 29.90 | | 25.31 | 38.62 |
| R*_merge_* (%)^*^ | 0.09827 (1.033) | | 0.08522 (0.9069) | 0.05702 (1.211) |
| CC (1/2) ^*^ | 0.998 (0.691) | | 0.998 (0.626) | 0.998 (0.509) |
| Refinement |  | |  |  |
| R*_work_*/R*_free_* (%) | 17.0 / 21.7 | | 16.7 / 20.7 | 18.5 / 22.6 |
| No. atoms | 5,368 | | 5,517 | 8,894 |
| Protein | 5,005 | | 4,984 | 8,443 |
| Ligands | 137 | | 192 | 132 |
| Solvent | 312 | | 455 | 395 |
| B-factor (Å^2^) | 43.95 | | 36.09 | 53.94 |
| R.m.s deviations |  | |  |  |
| Bond lengths (Å) | 0.013 | | 0.012 | 0.011 |
| Bond angles (°) | 1.26 | | 1.08 | 1.33 |
| Ramachandran (%) |  | |  |  |
| Favored (%) | 97.24 | | 97.56 | 97.93 |
| Outliers (%) | 0.00 | | 0.00 | 0.00 |

^*^Statistics for the highest-resolution shell are shown in parentheses.
